# Ventral tegmental area glutamate neurons co-release GABA and promote positive reinforcement

**DOI:** 10.1038/ncomms13697

**Published:** 2016-12-15

**Authors:** Ji Hoon Yoo, Vivien Zell, Navarre Gutierrez-Reed, Johnathan Wu, Reed Ressler, Mohammad Ali Shenasa, Alexander B. Johnson, Kathryn H. Fife, Lauren Faget, Thomas S. Hnasko

**Affiliations:** 1 Department of Neurosciences, University of California, San Diego, La Jolla, California 92093, USA; 2 Biomedical Sciences Graduate Program, University of California, San Diego, La Jolla, California 92093, USA; 3 Neuroscience Graduate Program, University of California, San Diego, La Jolla, California 92093, USA

## Abstract

In addition to dopamine neurons, the ventral tegmental area (VTA) contains GABA-, glutamate- and co-releasing neurons, and recent reports suggest a complex role for the glutamate neurons in behavioural reinforcement. We report that optogenetic stimulation of VTA glutamate neurons or terminals serves as a positive reinforcer on operant behavioural assays. Mice display marked preference for brief over sustained VTA glutamate neuron stimulation resulting in behavioural responses that are notably distinct from dopamine neuron stimulation and resistant to dopamine receptor antagonists. Whole-cell recordings reveal EPSCs following stimulation of VTA glutamate terminals in the nucleus accumbens or local VTA collaterals; but reveal both excitatory and monosynaptic inhibitory currents in the ventral pallidum and lateral habenula, though the net effects on postsynaptic firing in each region are consistent with the observed rewarding behavioural effects. These data indicate that VTA glutamate neurons co-release GABA in a projection-target-dependent manner and that their transient activation drives positive reinforcement.

## Abstract (web summary)

Ventral tegmental area (VTA) is involved in reward behaviours, but the precise contribution of VTA glutamatergic neurons to this process is not known. Here the authors show that phasic but not sustained optogenetic stimulation of VTA glutamatergic neurons is rewarding and involves co-release of GABA.

The ventral tegmental area (VTA) is a heterogeneous brain region that serves as a critical hub in the control of motivated behaviours. VTA neurons send dense dopamine projections to the nucleus accumbens (NAc) and olfactory tubercle, and more modest projections to other limbic regions including the amygdala and prefrontal cortex (PFC)^1^. Dopamine neurons tend to fire in response to rewards and reward-predicting cues, and dopamine signalling has profound impacts on reward-seeking and behavioural reinforcement through activation of G protein-coupled dopamine receptors. But VTA neurons also release other signalling molecules, including the major inhibitory and excitatory neurotransmitters in the brain γ-aminobutyric acid (GABA) and glutamate^2^,^3^,^4^,^5^,^6^,^7^,^8^,^9^,^10^,^11^; though a systems-level understanding of the function of fast postsynaptic signals provoked by GABA or glutamate co-release has remained elusive^12^,^13^.

Although the co-release of GABA or glutamate with dopamine is more common than originally suspected, significant populations of VTA neurons are devoid of dopamine markers but express markers indicative of GABA or glutamate release. Indeed, the canonical GABA markers, glutamic acid decarboxylase and the vesicular GABA transporter (VGAT) are virtually absent from immunochemically identified dopamine neurons in the VTA, but present in 20–30% of all VTA neurons^14^,^15^,^16^,^17^. VTA GABA neurons are important targets of drugs of abuse, and plasticity at inhibitory synapses to and from VTA contribute to maladaptive behaviours common in drug addiction^18^,^19^,^20^. Optogenetic approaches have recently demonstrated that VTA GABA neurons are responsive to reward-predicting cues and to aversive stimuli^21^,^22^; and their activation is sufficient to disrupt reward consumption or induce avoidance behaviour^23^,^24^.

Systematic efforts to characterize VTA glutamate neurons have begun only recently, in part, because of their relatively delayed discovery that required the *in situ* detection of mRNA encoding the vesicular glutamate transporter 2 (VGLUT2.)^25^,^26^. Also, some ambiguity persists regarding the prevalence of VGLUT2 in the VTA, with one quantitative stereological assessment indicating VGLUT2^+^ neurons comprise just 2–3% of the rat VTA (ref. 14). However, VGLUT2^+^ neurons are concentrated in medial VTA subregions where they can outnumber dopamine neurons; indeed, one study indicates that VGLUT2^+^ neurons represent ∼35% of NAc-projecting neurons in VTA, as well ∼66% of those projecting to PFC (ref. 27). These findings are consistent with observations that most if not all neurons in the NAc receive glutamate input from VTA (refs 4, 10, 28) and multiple other lines of evidence suggesting that VGLUT2^+^ VTA neurons may prove markedly more prevalent than initially surmised^29^,^30^,^31^,^32^,^33^.

Recent reports have used optogenetics to suggest that VTA neurons may convey either reward^34^ or aversion^35^,^36^ signals depending on local connectivity or projection target. In this report we selectively targeted VTA VGLUT2^+^ neurons using optogenetics and showed that brief photostimulation of cell bodies or three major terminal fields was sufficient to induce positive reinforcement in instrumental behavioural assays; but that their sustained stimulation was less preferred and could even manifest as apparent behavioural avoidance. These data are in stark contrast to the effects produced by VTA dopamine neuron stimulation, which induced reward responses across a wide range of behavioural assays and stimulation parameters. We found also that a subset of VGLUT2^+^ VTA neurons projecting to the ventral pallidum (VP) or lateral habenula (LHb) co-release glutamate and GABA. However, in the absence of other inputs and congruent with the behavioural reinforcement induced by short stimulus trains, train activation of VGLUT2^+^ VTA inputs to VP were net excitatory, but net inhibitory in the LHb. We thus propose that transient activation of VGLUT2^+^ VTA neurons promotes behavioural reinforcement through their ability to differentially excite or inhibit postsynaptic cells dependent on projection target, but that the effects of sustained activity are less preferred.

## Results

### Optogenetic manipulation of VGLUT2^+^ VTA neurons

**Figure 1:**
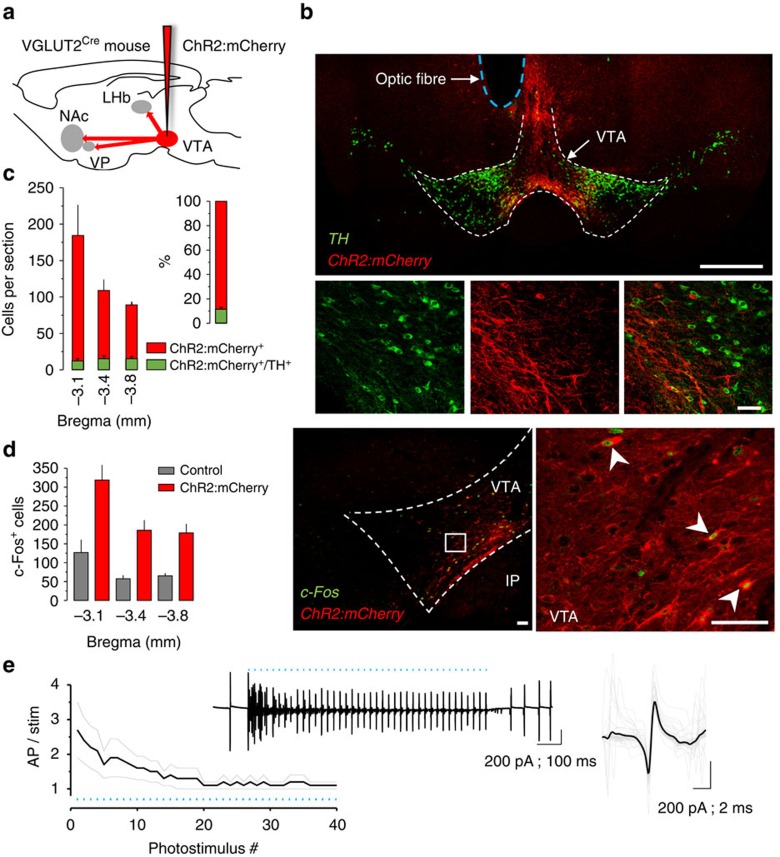
Functional expression of ChR2 in VGLUT2^+^ VTA neurons. (**a**) Schematic illustrating strategy for selective expression of ChR2:mCherry in VGLUT2^+^ VTA neurons. (**b**) ChR2:mCherry expression (red) restricted to medial VTA in sections stained for TH (green); scales, 500 μm (top), 50 μm (bottom). (**c**) VTA soma clearly labelled for ChR2:mCherry were counted in each of three coronal planes and assessed for co-labeling with TH. Inset shows fraction of all ChR2:mCherry-labelled cells that co-label for TH. (**d**) Quantification and example images of c-Fos^+^ cells (green) in the VTA of control and ChR2:mCherry-expressing mice following photostimulation; *P*<0.01 (**e**) Cell-attached recordings from ChR2:mCherry^+^ neurons in VTA demonstrate photostimulus-induced spike entrainment and spike fidelity ≥1 for 1 s at 40 Hz. Insets show example train and mean evoked action potential waveform; blue marks indicate 5-ms light pulses.

To selectively manipulate VGLUT2^+^ neurons in the VTA, we stereotactically injected a Cre recombinase-dependent Adeno-associated viral vector engineered to express the fusion protein Channelrhodopsin-2:mCherry (AAV1-EF1α-DIO-ChR2:mCherry). The vector was infused unilaterally into the medial VTA of *Slc17a6*^*IRES-Cre*^ (VGLUT2-Cre) mice^37^, where VGLUT2^+^ neurons are most abundant (Fig. 1a). A subset of sections were co-stained for the dopamine neuron marker tyrosine hydroxylase (TH); 11.4±1.5% of clearly labelled ChR2:mCherry^+^ cell bodies co-labelled with TH (*n*=4 mice; Fig. 1b-1c), consistent with previous reports showing overlap between VGLUT2^+^ and TH^+^ cells^3^,^25^,^27^,^30^,^35^,^38^. Mice used in behavioural experiments were also implanted with chronic indwelling optic fibres dorsal to the medial VTA; implant sites were verified by histology after behavioural experiments (Supplementary Fig. 1). Subsets of mice were also processed to demonstrate that photostimulation could induce a significant increase in the number of c-Fos labelled cells in the VTA (Fig. 1d).

To characterize the membrane properties of ChR2:mCherry-labelled cells and to demonstrate optogenetic control over their activity, whole-cell or cell-attached recordings were made from acute brain slices. Recordings showed that trains of photostimuli were sufficient to evoke action potentials with spike fidelity ≥1 up to at least 40 pulses delivered at 40 Hz (Fig. 1e). Similar to previous reports using BAC transgenic VGLUT2-EGFP mice^28^, ChR2:mCherry-labelled cells in VTA were typically spontaneously active with firing rates of 5.2±1.2 Hz (Supplementary Fig. 2).

### Activation of VGLUT2^+^ VTA soma or terminals is reinforcing

**Figure 2:**
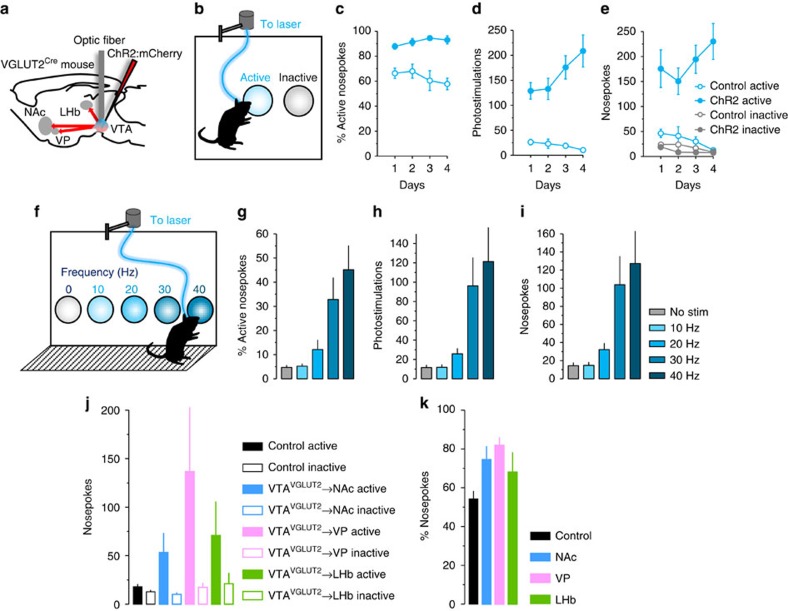
Operant discrimination shows photostimulation of VGLUT2^+^ VTA neurons is positively reinforcing. (**a**) Schematic illustrating strategy for selective expression and photostimulation of ChR2 in VGLUT2^+^ VTA neurons. (**b**) Schematic illustrating 2-nosepoke discrimination task where responding on the active nosepoke triggers 80 pulses at 40 Hz. (**c**) ChR2-expressing mice but not control mice develop a preference for the active nosepoke; *P*<0.001. (**d**) ChR2-expressing mice trigger more photostimuli than control littermates; *P*<0.001. (**e**) ChR2-expressing mice make more active nosepokes than controls; *P*<0.001. (**f**) Schematic illustrating 5-choice nosepoke task where each of 5 nosepokes are coupled to 1-s photostimulus at varying frequency. Frequency-response histograms reveal ChR2-expressing mice display an (**g**) ascending preference for faster stimulation; *P*<0.001, (**h**) trigger more stimuli; *P*<0.001, and (**i**) have higher response rates at nosepoke holes coupled to faster photostimulation; *P*<0.001 compared with responding on control nosepoke hole (for no stimulation). (**j**) Mice expressing ChR2 in VGLUT2^+^ VTA neurons were implanted instead with fibres in each of the NAc, VP or LHb to target presynaptic terminals. When placed on a restricted feeding schedule and assessed using the 2-nosepoke instrumental task, mice displayed an increase in responding; *P*<0.01 and a (**k**) preference for the active nosepoke compared with controls. Data in **j** and **k** represent average responses over 5 days.

Because optogenetic stimulation of dopamine neurons is sufficient to drive positive behavioural reinforcement^39^,^40^, while activation of VTA GABA neurons can oppose reward^23^,^24^, we tested whether photostimulation of VGLUT2^+^ VTA neurons can suffice as a reinforcer using operant behavioural tasks (Fig. 2a,b). Using a 2-hole nosepoke-operant discrimination assay where an active nosepoke triggered optical stimulation of VGLUT2^+^ VTA neurons, mice demonstrated single-session discrimination for the active nosepoke (Fig. 2c–e, Supplementary Video 1). Responding persisted over the course of 4 days and control littermates lacking ChR2:mCherry expression showed no discrimination and minimal responding overall. Similar results were observed using a different VGLUT2-Cre BAC transgenic mouse line^41^ (Supplementary Fig. 3) or with a 2-bottle choice assay in water-deprived mice, even when optical stimulation was placed in competition with sucrose solution (Supplementary Fig. 4). Subsequent exposure to a 5-hole nosepoke apparatus, such that each nosepoke was coupled to light delivery at frequencies varying from 0–40 Hz (Fig. 2f), demonstrated that mice displayed a strong preference for light delivered at faster frequencies, with maximal responding for 40 Hz, the highest frequency tested (Fig. 2g–i).

Several brain regions are densely innervated by VGLUT2^+^ VTA neurons; including the medial shell of the NAc, VP, and LHb (refs 28, 30). To determine whether individual VTA neurons might target more than one of these non-contiguous structures we injected different coloured fluorescent retrobeads into pairs of these brain regions (that is, NAc/VP, NAc/LHb and VP/LHb; Supplementary Fig. 5a). We found many retrobead-labelled cells in the VTA, but only a small fraction (<1%) co-localized for both colours (Supplementary Fig. 5b–d). These data indicate that separate populations of VTA neurons, including VTA glutamate neurons, target each of these structures. Changes in neural activity in each of these regions could support behavioural reinforcement observed following cell-body stimulation (Fig. 2), but with different expected valences. For example, increased excitatory drive to the NAc or VP can drive positive reinforcement^42^,^43^, but activation of the LHb is negatively reinforcing^44^. If release of glutamate at these varied efferent targets is expected to have opposing effects on behavioural reinforcement, why does photostimulation of VGLUT2^+^ VTA cell bodies produce robust positive reinforcement?

Surprisingly, photostimulation of VGLUT2^+^ VTA terminal fields in each of the NAc, VP and LHb initially failed to induce positive reinforcement on the 5-nosepoke and 2-bottle choice tasks (Supplementary Fig. 6a–c). However, when the mice were placed on a restricted feeding schedule and exposed to the 2-hole nosepoke-instrumental task we observed modest but significant increase in responses for the active hole (Fig. 2j,k). These results indicate that activation of VGLUT2^+^ VTA terminals in each of these regions is capable of serving as a reinforcer, at least under certain conditions. The less robust reinforcement may relate to a reduced ability to efficiently recruit terminals on photostimulation, indicate that synchronous activation of multiple targets is a more potent reinforcer, or that the reinforcing effects are state dependent.

Because a subset of VGLUT2^+^ neurons co-localize with TH (Fig. 1b,c) and co-release dopamine in the NAc (refs 3, 4), the coincident release of dopamine is likely to contribute at least partly to the reinforcing properties of stimulating VGLUT2^+^ VTA neurons or NAc terminals. As an initial test of the relative contribution of photo-evoked dopamine to the observed behavioural reinforcement, we repeated the experiments following systemic administration of dopamine D1 receptor (D_1_R), dopamine D2 receptor (D_2_R) or combined antagonists. We found that at doses that can inhibit dopamine-dependent reinforcement^45^,^46^, neither D_1_R (Supplementary Fig. 7a) nor D_2_R (Supplementary Fig. 7b) antagonism blunted responding on the two-bottle choice assay. Similar results were obtained when both antagonists were provided jointly using a counter-balanced design in mice stably responding on the two-hole nosepoke instrumental reinforcement assay (Supplementary Fig. 7c). Importantly, this treatment significantly attenuated responding for photostimulation of DAT^+^ VTA neurons (Supplementary Fig. 7d). These data suggest that self-stimulation of VGLUT2^+^ VTA neurons is a potent reinforcer relatively resistant to pharmacological blockade of coincident photo-evoked dopamine.

### Projection target-specific GABA/glutamate co-release

**Figure 3:**
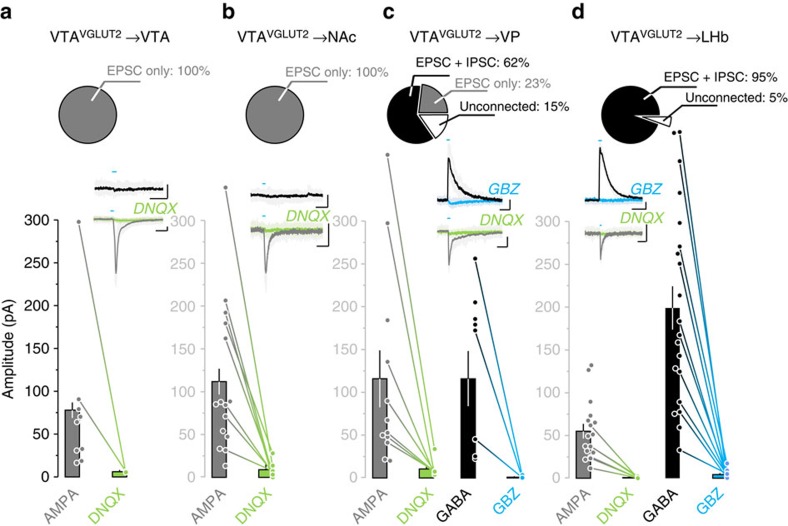
VGLUT2^+^ VTA terminals co-release glutamate and GABA in projection-target restricted fashion. Single-pulse (5-ms, blue dashes) photostimulation of (**a**) local VTA collaterals or (**b**) terminals in the NAc triggered DNQX-sensitive EPSCs (*V*_h_=−60) but no IPSCs (*V*_h_=0). Photostimulation of VGLUT2^+^ VTA terminals in (**c**) VP led to EPSCs in all connected cells, but also gabazine-sensitive IPSCs. All connected cells in the (**d**) LHb displayed both EPSCs and IPSCs. Pie charts show the percentage of neurons that showed light-triggered EPSCs only (grey), both EPSCs and IPSCs (black), or no currents (white). Bar graphs show peak amplitude of E/IPSCs, with points representing individual cells pre- and post- drug application; representative traces are inset. Scales, 50 pA, 50 ms.

To investigate alternate mechanisms by which VGLUT2^+^ VTA neurons may induce behavioural responses we assessed the postsynaptic effects induced by activation of VGLUT2^+^ VTA neurons locally within the VTA and from distal terminals in NAc, VP and LHb. Using whole-cell voltage clamp we identified and recorded from mCherry-negative VTA neurons. Photostimulation of VTA cell bodies led to DNQX-sensitive excitatory postsynaptic currents (EPSCs) in all ChR2:mCherry-negative VTA neurons that we recorded from (Fig. 3a), consistent with previous reports suggesting local excitatory connections within the VTA^34^,^47^. Photostimulation of VGLUT2^+^ VTA inputs to medial NAc shell (Fig. 3b), VP (Fig. 3c) and LHb (Fig. 3d) also revealed high rates of connectivity and EPSCs were present in all connected cells. However, photostimulation of VGLUT2^+^ VTA terminals additionally revealed gabazine-sensitive inhibitory postsynaptic currents (IPSCs) in the VP and LHb, but not in VTA or NAc (Fig. 3). These IPSCs had short delays consistent with monosynaptic connections (Table 1), were unaffected by bath application of DNQX and APV, but were blocked by tetrodotoxin and recovered in 4-AP (Supplementary Fig. 8), confirming their monosynaptic nature. Importantly, when the experiment was performed using *Slc32a1*^*IRES-Cre*^ (VGAT-Cre) mice, both IPSCs and EPSCs were detected in the LHb and VP (Supplementary Fig. 9), but only IPSCs were found in NAc and VTA; indicating that ectopic Cre expression is unlikely to account for these findings. Rather, these results suggest that a subset of VGLUT2^+^ neurons in the VTA co-release GABA in a projection-target specific manner.

**Figure 4:**
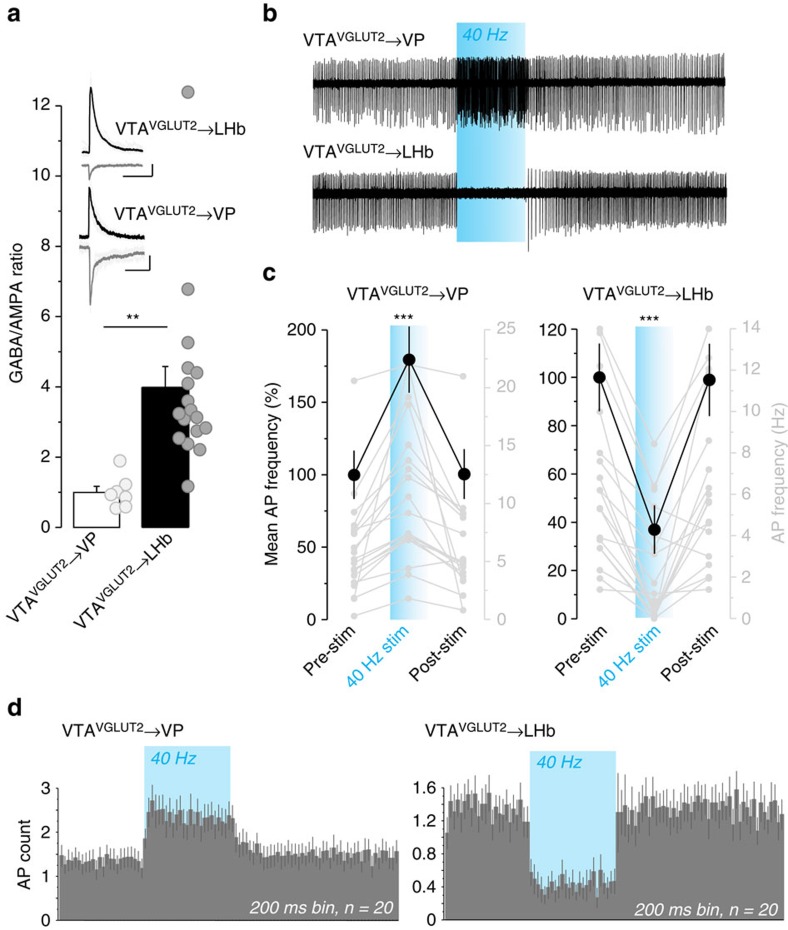
VGLUT2^+^ VTA terminals are net excitatory in VP but inhibitory in LHb. (**a**) GABA/AMPA ratios calculated from voltage-clamp recordings at *V*_h_=0/*V*_h_=−60 suggest the effects of GABA are predominant in the LHb compared with VP. The value of the GABA/AMPA ratio for each individual recorded neuron is represented by the grey rounds. The top insets show representative traces of GABA IPSC (black) and glutamate EPSC (grey); **P<0.01, scales 50 pA, 50 ms. (**b**) Representative cell-attached traces in the VP (top) or LHb (bottom) showing firing before, during (blue), and after 40-Hz 5-s photostimulation. (**c**) Photostimulation of VGLUT2^+^ VTA terminals led to a consistent increase in firing in the VP, but a decrease in the LHb. The left (black) axis and plots show mean±s.e.m. firing rates 5-s before, during (blue background), and after photostimulation; the right (grey) axis and plots show firing rates (Hz) for the individual neurons. Though we occasionally observed a transient rebound effect following light off, the firing frequency in the 5-s bin after the stimulation was not significantly different from the 5-s bin prior; ***P<0.001. (**d**) Data from C in 200-ms bins shows that the excitatory effects on VP and inhibitory effects on LHb persist for the duration of the 5-s stimulus train.

To compare the relative impact of the GABA and glutamate signals on postsynaptic cells across regions, we first calculated the ratios of outward currents recorded at *V*_h_=0 mV to inward currents at *V*_h_=−60 mV in the VP and LHb in response to photostimulation of VGLUT2^+^ VTA terminals. In the VP this GABA/AMPA ratio was equal to ∼1, but was ∼4 in the LHb (Fig. 4a), suggesting that the relative effects of GABA co-release may be stronger in the LHb compared to VP. We next tested whether the synaptic effects of terminal photostimulation could be sustained at high frequency. First, to assess how photostimulation of VGLUT2^+^ VTA inputs influences firing of postsynaptic cells, we made cell-attached recordings, taking advantage of the fact that VP and LHb neurons tend to fire spontaneously in acute slice. Photostimulation (40 Hz, 5 s) of VGLUT2^+^ VTA terminals in VP led to a consistent increase in firing in postsynaptic cells (Fig. 4b,c) that persisted for the duration of the 5-s photostimulus trains (Fig. 4d). On the other hand, photostimulation of VGLUT2^+^ VTA terminals in the LHb produced a persistent decrease in the firing rate of postsynaptic cells (Fig. 4b–d). Together, these data suggest that the net effects of terminal stimulation in the VP are excitatory, but inhibitory in the LHb.

Although the effects of terminal photostimulation on postsynaptic firing demonstrate an ability of these synapses to maintain functional transmission at 40 Hz for at least 5 s, we noted that sustained photostimulation led to marked depression at each synapse examined and for both EPSCs and IPSCs (Supplementary Fig. 10). The size of the second response (paired-pulse ratio) with inter-stimulus intervals ranging from 500 to 25 ms (thta is, 2–40 Hz) showed mean reductions (that is, paired-pulse depression) in amplitude of up to 84%; with shorter inter-stimulus interval and longer stimulus trains producing cumulatively greater depression (Supplementary Fig. 10). These data suggest a high probability of release, consistent with previous results examining glutamate co-release from dopamine terminals in the NAc (ref. 48), and could indicate that sustained stimulation would produce effects functionally distinct from brief stimulation.

### Brief stimulation of VGLUT2^+^ VTA neurons is preferred

**Figure 5:**
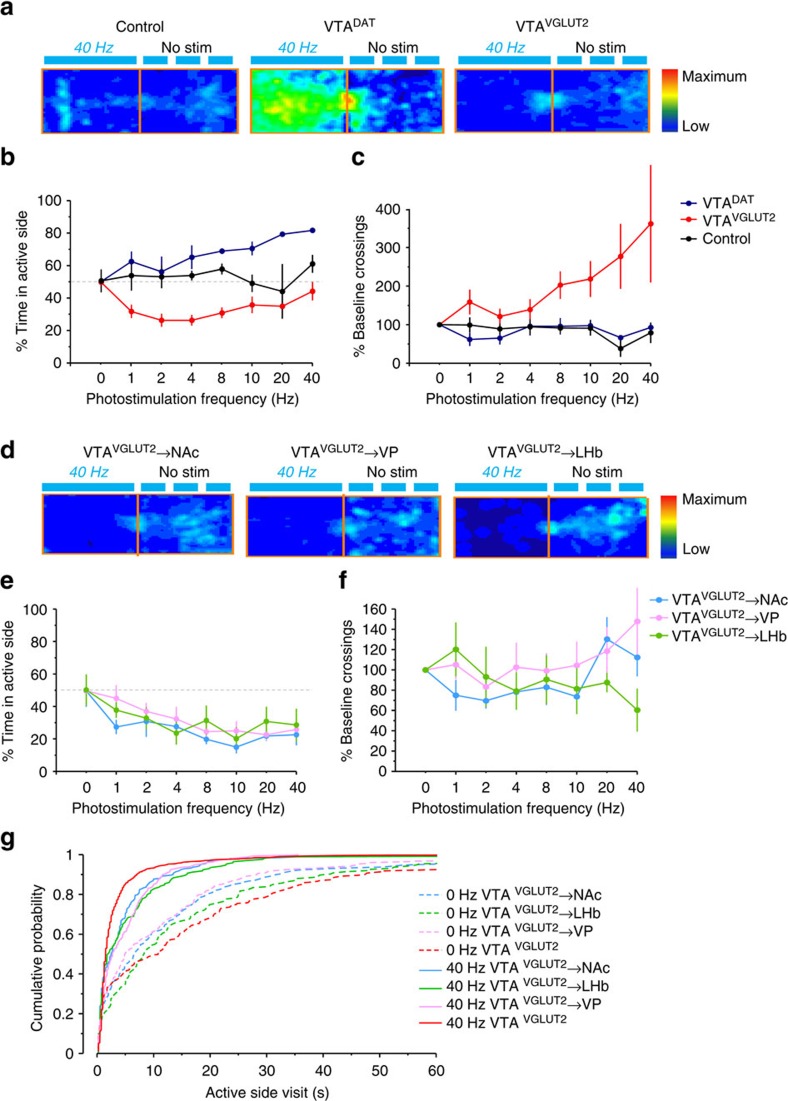
Mice spend less time but make more entries into compartments paired with stimulation of VTA glutamate neurons. (**a**) Example location heat maps of relative times spent in 20 min RTPP 20-min RTPP assay, comparing photostimulation of ChR2-negative controls, DAT^+^ dopamine, or VGLUT2^+^ glutamate neurons in the VTA. (**b**) Per cent of time spent on the active side reveals a frequency-dependent increase in time spent on the active side for dopamine neuron stimulation *P*<0.01; low frequency stimulation of VTA glutamate neurons led to a significant reduction in time spent on active side, but this effect was mitigated with higher frequency. (**c**) Stimulation of VGLUT2^+^ VTA neurons led to a frequency-dependent increase in the number of crossings between active and inactive sides; *P*<0.05. (**d**) Example heat maps comparing photostimulation of VGLUT2^+^ VTA terminals in the NAc, VP or LHb. (**e**) Terminal stimulation leads to an apparent frequency-dependent place avoidance; *P*<0.001, but with (**f**) no decrease in crossings. (**g**) Significant shifts in the distributions toward shorter visits is consistent with the possibility that apparent avoidance reflects preference for brief burst stimulation rather than overt avoidance/aversion; *P*<0.001.

To test the effects of sustained stimulation, we employed several additional behavioural tasks and compared the effects of stimulating VGLUT2^+^ VTA cell bodies with their terminal stimulation, and also to the effects of stimulating DAT^+^ VTA dopamine cell bodies. First, we used an ascending stimulus frequency (0–40 Hz, increasing across days) real-time place procedure (RTPP), where mice were provided free access to two compartments, one of which was coupled to photostimulation. Note that in the RTPP, mice control the duration of stimulation by exiting the photostimulus-paired (that is, ‘active') compartment. When photostimulation was used to activate DAT^+^ VTA dopamine neurons we, as expected, observed a frequency-dependent increase in time spent in the active compartment, though no change in the number of side changes (that is, ‘crossings') (Fig. 5a–c). Surprisingly, when we performed the same experiment to stimulate VGLUT2^+^ VTA neurons, we observed an apparent place avoidance at low frequencies that was mitigated at higher frequencies (Fig. 5a,b). However, this was accompanied by a frequency-dependent appetitive increase in the number of crossings (Fig. 5c; Supplementary Video 2) and a significant shift in the distribution towards shorter active-side visits (Fig. 5g). Mice thus appeared to titrate the amount of time spent in the active compartment to maximize the number of brief photostimuli received. Photostimulation of VGLUT2^+^ VTA terminals showed a similar apparent avoidance for the active side and distribution shift; although there was a tendency towards increased crossings at higher frequencies, they were not significant (Fig. 5d–g). Similar results were observed using bilateral stimulation and a more typical single frequency RTPP design (Supplementary Fig. 6d–h).

**Figure 6:**
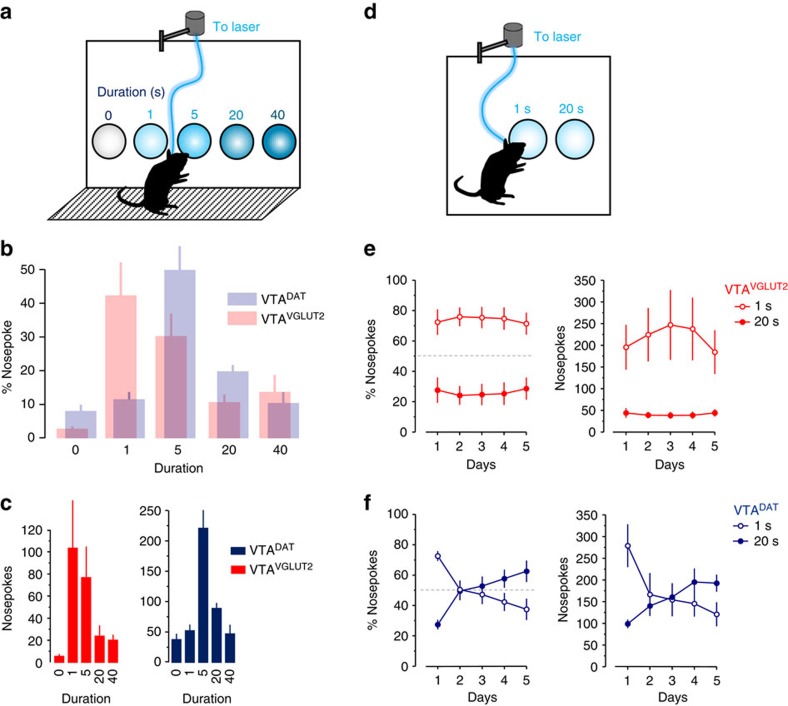
Mice prefer brief stimulation of VTA glutamate but sustained stimulation of VTA dopamine neurons. (**a**) Using a 60-min 5-nosepoke choice assay with 40-Hz stimulation coupled to different stimulus durations, (**b**) photostimulation of VGLUT2^+^ VTA neurons produces a preference for short over longer stimulus duration compared with DAT^+^ VTA neurons; *P*<0.01. (**c**) Nosepoke responses for the varying stimulus durations; note that only the final day is shown here, but all days are displayed in Supplementary Fig. 10. (**d**) Using a 2-nosepoke choice assay with 40-Hz stimulation coupled to 1- or 20-s photostimulation, (**e**) mice expressing ChR2 in VGLUT2^+^ VTA neurons displayed a significant preference for shorter stimulation *P*<0.01. (**f**) In comparison, stimulation of DAT^+^ VTA neurons leads to the development of a preference for longer stimulus durations; *P*<0.001.

The apparent avoidance observed in the RTPP seems to conflict with the strong positive reinforcement observed following VGLUT2^+^ VTA cell-body stimulation (Fig. 2 and Supplementary Figs 3 and 4), as well as with the positive reinforcement observed when stimulating their terminals in the instrumental task (Fig. 2j,k), and the absence of avoidance by two-bottle choice (Supplementary Fig. 6c). However, the increase in number of crossings in the RTPP (Fig. 5c) is consistent with the hypothesis that the mice are engaging in an appetitive behaviour, perhaps titrating time spent to achieve preferred short photostimulus trains. To directly compare short versus long stimulus trains, we employed 5- and 2-hole nosepoke instrumental choice tasks, now using constant frequency (40 Hz) with variable stimulus duration assigned to each nosepoke (Fig. 6a,d). Following 3 days of exposure to a 5-nosepoke apparatus comparing 0–40 s of 40 Hz stimulation (0, 40, 200, 800 or 1,600 pulses; Supplementary Fig. 11), we observed a significant difference in responding for DAT^+^ versus VGLUT2^+^ VTA stimulation. Mice showed a clear preference for brief (≤5 s) stimulation trains when coupled to VGLUT2^+^ VTA neuron stimulation compared with a preference for longer (≥5 s) trains when coupled to DAT^+^ VTA neuron stimulation (Fig. 6a–c). In a second experiment, we tested mice over 5 days on a 2-nosepoke task comparing 1- versus 20-s stimulation at 40 Hz (40 or 800 pulses). Mice receiving stimulation of VGLUT2^+^ VTA neurons showed a stable preference for the nosepoke coupled to 1-s trains (Fig. 6e). In contrast, following an initial preference for the shorter stimulus train, mice receiving stimulation of DAT^+^ neurons ultimately developed a preference for the nosepoke coupled to longer stimulation (Fig. 6f). The results indicate that transient photostimulation of VGLUT2^+^ VTA cell bodies is preferentially reinforcing in operant tasks, and this preference for short intermittent trains of stimuli may contribute to an apparent place avoidance by RTPP.

Consistent with the interpretation that prolonged activation of VGLUT2^+^ VTA neurons is neither potently rewarding nor aversive, we found that their sustained inhibition using Halorhodopsin did not alter behaviour in the RTPP, whereas inhibition of DAT^+^ VTA neurons led to strong avoidance (Supplementary Fig. 12). These data further distinguish the two cell populations and suggest that while tonic activity of VTA dopamine neurons provides a value signal, VTA glutamate neurons are either not tonically active or their tonic activity lacks such valence.

## Discussion

The identification of the VGLUTs provided definitive markers of glutamate-releasing neurons^49^, and led to the subsequent discovery of VGLUT2-expressing neurons in the VTA^25^,^26^. Much of the initial work targeting this population was aimed at deciphering the function of VGLUT2 in dopamine neurons; and conditional disruption of VGLUT2 from dopamine neurons resulted in reductions in psychostimulant-induced locomotion and evoked dopamine release^3^,^50^,^51^,^52^. However, the majority of VGLUT2^+^ neurons detected in mouse VTA are not dopaminergic^29^, suggesting that the conditional knockout of VGLUT2 selectively from dopamine neurons left glutamate signalling from VTA neurons largely intact.

In this report, we used VGLUT2-Cre mice and optogenetics to target VGLUT2^+^ VTA neurons directly, irrespective of their ability to co-release other signalling molecules. We found that photostimulation of VGLUT2^+^ projection neurons served as a potent reinforcer on operant assays, consistent with another recent report^34^. Though this approach also recruits dopamine release and we cannot deny that dopamine signalling contributes to the behavioural results we observed, multiple lines of evidence suggest that VTA glutamate neurons impact reward in a manner that is distinct from dopamine neurons. First, we found only 11% of ChR2:mCherry-labelled neurons were TH^+^ in VGLUT2-Cre mice. Second, the ability of VGLUT2^+^ VTA neurons to directly excite most neurons in the NAc and VP while inhibiting LHb neurons (via GABA co-release) are each plausible mechanisms to drive behavioural reinforcement^42^,^43^,^44^,^53^. Third, photostimulation (or photoinhibition) of DAT^+^ dopamine versus VGLUT2^+^ glutamate neurons in the VTA led to divergent behavioural patterns, in particular suggesting that sustained high-frequency stimulation of VGLUT2^+^ neurons is less preferred compared with brief stimulus trains. Fourth, operant responding for VGLUT2^+^ VTA neuron stimulation is resistant to the effects of dopamine receptor antagonists at a dose that significantly reduced responding for DAT^+^ VTA neuron stimulation.

Despite the profound self-stimulation induced in the operant assays, we were unable to reliably induce a place preference using the RTPP for photostimulation of VGLUT2^+^ VTA neurons. Rather, terminal stimulation as well as low-frequency stimulation of cell bodies led to a pronounced decrease in the time spent in the active compartment, potentially indicating that their prolonged activation conveys an aversive signal. Others too have reported that 20 Hz stimulation of VGLUT2^+^ VTA terminals in the LHb or NAc induced avoidance using RTPP procedures^35^,^36^,^54^. Moreover, Qi *et al*.^35^ found that mice trained to lever press for food preferred a lever not paired to photostimulation (20 Hz) of VGLUT2^+^ terminals in the NAc, and preferred to spin a wheel that turned off ongoing photostimulation of the same. These data have been interpreted to indicate that VGLUT2^+^ VTA neurons signal reward via local activation of other VTA neurons, but signal aversion via distal activation of NAc interneurons or LHb projection neurons^34^,^35^; though it is not clear whether local connections are made by a discrete population of excitatory VTA interneurons, collateralization of VTA projection neurons or both. However, excitatory inputs to NAc from hippocampus, amygdala and PFC have been shown to drive positive reinforcement^42^,^55^. The excitatory input to NAc from VTA has the added ability to co-release dopamine^56^ and data presented here and published^4^,^7^,^10^,^28^,^35^ suggest that VTA glutamate inputs broadly target essentially all NAc shell cell types (though may vary by synaptic strength/incidence). Similarly, our data as well as work published by others^57^ suggest that the dominant effects of activating VGLUT2^+^ VTA terminals in the LHb is inhibitory, which is rather associated with positive reinforcement^44^,^53^. Together these data suggest that VGLUT2^+^ VTA projections are likely to play a faciliatory role in positive reinforcement. Indeed, we found that activation of VGLUT2^+^ VTA terminals in the LHb, VP and NAc were each sufficient to drive self-stimulation in an operant assay when animals were placed on a restricted feeding schedule. Interestingly, water-restricted mice did not show a clear preference for a sipper coupled to terminal photostimulation in the two-bottle choice assay; though importantly no avoidance/aversion was detected either. Because these observations were made in multiple cohorts of mice, including mice individually tested in both operant and RTPP assays, we suspect that the apparent avoidance for cell-body or terminal stimulation that we and others observed does not represent aversion.

Why then might stimulation of VGLUT2^+^ VTA neurons induce apparent avoidance behaviour in the RTPP task? The first clue came from our observation that mice exhibited the unusual behaviour of repeatedly making brief entries into the stimulus-paired compartment (Supplementary Video 2), an apparent appetitive behaviour quantified by an increasing number of side crossings. This observation is in contrast to the effects of photostimulation of VTA dopamine neurons which did not lead to more crossings. We hypothesized that the mice titrated their exposure to increase the number of relatively brief stimulus epochs; thus the decrease in time spent on the active side, rather than indicating aversion associated with VGLUT2^+^ VTA neuron stimulation, reflects a preference for transient intermittent over more sustained stimulus trains. Indeed, when directly tested to determine a preferred stimulus train length using operant procedures, mice receiving VGLUT2^+^ VTA stimulation showed a clear preference for shorter duration trains, again in notable contrast to the effects of VTA dopamine neuron stimulation. These data suggest that the initial effects of stimulating VGLUT2^+^ VTA neurons are reinforcing, but rapid accommodations in signalling mitigate or extinguish the initial effect. Indeed, we observed pronounced short-term depression of both EPSCs and IPSCs following photoactivation of VGLUT2^+^ VTA terminals in each of the NAc, VP and LHb. Combined with their ability to co-release glutamate and GABA, such properties may allow these neurons to encode distinct types of temporally dynamic reward-related information; for example, satiation where an initial increase in VGLUT2^+^ VTA neuron activity could encode reward, but the reward signal loses potency or even reverses valence as activity is sustained.

Dopamine neurons projecting to NAc, striatum, PFC and other regions have been shown capable of co-releasing either glutamate or GABA^2^,^4^,^5^,^6^,^7^,^8^,^10^,^15^. The widespread co-release of fast excitatory or inhibitory signals along with the slower neuromodulatory dopamine signal is consistent with the idea that dopamine neurons also encode different types of information over different timescales^13^,^58^, with potentially important implications for the role of dopamine neurons in reward learning and in psychiatric illness^59^. However, the normal physiological and functional roles of VTA glutamate neurons cannot be determined by optogenetic stimulation alone, which tests sufficiency, is subject to caveats, and may not reflect *in vivo* firing patterns. Future studies assessing loss of function in more complex behavioural assays and direct observations of neural activity during behaviour will provide valuable insight.

A subset of TH-Cre^+^ neurons in the VTA release GABA, but little or no dopamine or glutamate, from terminals in the LHb (ref. 9). Questions have been raised regarding the use of TH-Cre mouse lines to selectively target midbrain dopamine neurons, due to the potential for ‘ectopic' expression of TH transcript and Cre expression in neurons that contain little or no TH protein^54^,^60^. Such questions raise important issues about what markers constitute a specific class of VTA neuron, whether such markers are stable across development and in the adult, and the use of Cre-expressing mouse lines to target-specific cell types^61^,^62^. For these reasons we validated our findings using multiple Cre lines. To target VGLUT2^+^ neurons we used both knock-in^37^ and BAC transgenic VGLUT2-Cre lines^41^, finding comparable anatomical, electrophysiological and behavioural results. We also used VGAT-Cre knock-in mice to target VTA GABA neurons, and showed that photostimulation of terminals in the VP and LHb led to both the expected IPSCs, but also glutamate-mediated EPSCs.

Other groups have shown that the LHb receives input from neurons with the potential to co-release both glutamate and GABA. The input from the entopeduncular nucleus contains neurons that may release both glutamate and GABA from single vesicles, their photostimulation can drive place avoidance, and the relative ratio of inhibition to excitation may be subject to presynaptic regulation by anti-depressants^63^,^64^. Within VTA, a population of VGLUT2^+^ VTA neurons that project to the LHb co-express GABAergic and glutamatergic markers and single-pulse optogenetic stimulation led to both excitatory and inhibitory responses^57^. Conversely, VTA cells projecting to NAc rarely labelled for both GABA and glutamate markers^35^. Our data are consistent with such findings, but show that train stimulation of VGLUT2^+^ terminals in the LHb is reliably inhibitory and that that GABA/glutamate co-release from VTA neurons is projection-target dependent. Indeed, GABA co-release was also observed from VTA terminals in the VP where the net effect of sustained stimulation was instead reliably excitatory, but we found no evidence for monosynaptic GABA release from VGLUT2^+^ projections to NAc or from local VTA collaterals. Importantly, these physiological findings are consistent with the behavioural observations that follow from optogenetic stimulation of VGLUT2^+^ cell bodies; because the inhibition of the LHb or activation of the VP, NAc and VTA might each be predicted to drive positive reinforcement^1^,^42^,^43^,^44^,^53^.

It is important to note that each of the target structures (excepting possibly the LHb) contains multiple cell types which may differentially alter behaviours in response to VGLUT2^+^ VTA input, however, we find high rates of synaptic connectivity ranging from 85% in the VP to 100% of connected cells in the NAc and VTA. These data strongly suggest that inputs do not qualitatively discriminate by cell type, though further studies will be needed to determine if there exist differing rates of synaptic incidence or synaptic strength by postsynaptic cell type. Finally, our data suggests that NAc-, LHb- and VP-projecting VTA neurons, including glutamate neurons, rarely target more than one of these structures but rather represent non-overlapping populations of neurons within VTA, consistent with earlier work comparing other projection target combinations^65^. Future studies will be required to determine whether VTA neurons that release glutamate locally also project elsewhere or represent a population of excitatory interneuron, with important functional implications.

Though the phenomenon of glutamate and GABA co-transmission in the adult CNS has been observed for over a decade^58^,^66^; the concept remains perplexing. What is the purpose of presynaptic neurons transmitting such ‘mixed messages'? It is interesting to note that interneurons appear to be scarce within the LHb (refs 67, 68), suggesting GABA/glutamate co-release may be an alternate mechanism through which inputs can produce bidirectional effects. GABA/glutamate co-transmission may be homeostatic, consistent with the idea that the relative abundance of GABA and glutamate markers may be adaptive and under dynamic regulation in the presynaptic compartment^63^,^69^. However the net effect of glutamate/GABA co-release will also depend on the instantaneous excitability of the postsynaptic cell; and on the relative expression and trafficking of postsynaptic receptors which are also subject to dynamic regulation^70^,^71^,^72^. Understanding the mechanisms by which glutamate/GABA co-release is regulated in maladaptive states such as depression, anxiety and drug addiction will prove a fruitful area of future investigation. Our data provide important new insights into the role of a population of VTA glutamate neurons that differentially release multiple small-molecule transmitters across diverse efferent targets and can drive positive reinforcement.

## Methods

### Animals

Mice were bred at UCSD, group housed, and maintained on a 12 h light–dark cycle with food and water available *ad libitum* unless noted. Initial breeders were obtained from: *Slc17a6*^*tm2(cre)Lowl*^ (stock no: 016963), *Slc32a1*^*tm2(cre)Lowl*^ (stock no: 016962), *Slc6a3*^*tm1.1(cre)Bkmn*^ (stock no: 006660; The Jackson Laboratory) and BAC Tg. *Scl17a6-Cre* from Dr Ole Kiehn (Karolinska Institute). All mice were maintained fully back-crossed on to C57Bl/6, with the exception of *Slc32a1*^*tm2(cre)Lowl*^ which were maintained as homozygous C57Bl/6 × 129 Sv hybrids. Control mice included wild-type littermates receiving the same viral treatment and/or Cre^+^ littermates receiving treatment with a control viral vector as described below. Both male and female mice were included and all experiments performed in accordance with protocols approved by the University of California San Diego Institutional Animal Care and Use Committee.

### Stereotactic surgery

Mice (>4 weeks) were anaesthetized with isoflurane, placed in a stereotaxic frame (Kopf), and 300 nl of AAV1-EF1α-DIO-ChR2:mCherry (∼2 × 10^12^ genomes per ml, UNC gene therapy centre), AAV2-EF1α-DIO-mCherry (∼2 × 10^12^ genomes per ml, UNC gene therapy centre), AAV5-EF1α-DIO-EYFP (∼4 × 10^12^ genomes per ml, UNC gene therapy centre) or AAV5-EF1α-DIO-eNpHR3.0-EYFP (∼3.1 × 10^12^ genomes per ml, UNC) infused unilaterally into the left VTA (*x*=−0.3, *y*=−3.4, *z*=−4.4; mm relative to Bregma) at 100 nl min^−1^ (WPI UltraMicroPump) using custom made 30-gauge stainless (Plastics One, VA) injectors. The injection tip was left in place for an additional 10 min then slowly retracted. Mice used in behavioural experiments, following viral infusion, were implanted with an optic fibre constructed from 200-μm core multimode optical fibre (FT200EMT, Thorlabs) inserted into a ceramic ferrule (as described^73^) at one of the following coordinates: NAc (*x*=±0.8, *y*=1.2, *z*=−3.4), VP (*x*=±1.6, y=0, *z*=−5.0), LHb (*x*=−0.5, *y*=−1.8, *z*=−2.0) or VTA (*x*=−0.5, *y*=−3.4, *z*=−4.0). Fibres were stabilized in place using dental cement (Lang dental) secured by two skull screws (Plastics One). In eight cases mice were excluded following *post hoc* histological analyses where no native fluorescence was apparent in terminal regions. Animals were treated with analgesic Carprofen (Pfizer, 5 mg kg^−1^ s.c.) before and the day after surgery. Mice were monitored daily and allowed to recover from surgery ≥ 3 weeks before subsequent behavioural or physiological assays. Fluorescent retrobeads (Retrobeads IX – Red and Green, Lumafluor., 100–150 nl) were injected using a glass pipette or 30-gauge stainless injector at the following coordinates in C57BL/6 mice: NAc (*x*=−0.5, *y*=+1.7, *z*=−4.2; mm relative to Bregma), VP (*x*=−1.2, *y*=+0.38, *z*=−5.1; mm relative to Bregma) and LHb (*x*=−0.4, *y*=−1.7, *z*=−2.6; mm relative to Bregma). Fourteen days after injection mice were euthanized for histology.

### Histology

Mice were deeply anaesthetized with a mixture of ketamine (Pfizer, 10 mg kg^−1^ i.p.) and xylazine (LLOYD, 2 mg kg^−1^ i.p.) and transcardially perfused with 10–20 ml of phosphate-buffered saline (PBS) followed by at least 30 ml 4% paraformaldehyde at a rate of ∼6 ml min^−1^. Brains were extracted, incubated in 4% paraformaldehyde at 4 °C overnight then transferred to 30% sucrose solution for 48–70 h at 4 °C. Brains were snap frozen in chilled isopentane and stored at −80 °C. Sections (30 μm) were cut using a cryostat (CM3050S, Leica) and collected in PBS containing 0.01% sodium azide. For immunostaining, brain sections were gently rocked and washed 3 × 5 min in PBS, 3 × 5 min in PBS containing 0.2% Triton X-100 (PBS-Tx), and blocked with 4% normal donkey serum in PBS-Tx (block) for 1 h at room temperature. Sections were then incubated in primary antibodies (rabbit anti-TH, 1:2,000, Millipore AB152; sheep anti-TH, 1:2,000, Pel-Freez P60101-0; rabbit anti-c-Fos, 1:7,000, Calbiochem PC38; and/or Rat anti-substance P, 1:400, Millipore MAB356) in block at 4 °C overnight. Sections were rinsed 3 × 10 min with PBS-Tx and incubated in appropriate secondary antibodies (Jackson ImmunoResearch) conjugated to Alexa488 or Alexa647 fluorescent dyes (5 μg ml^−1^) for 2 h at room temperature. Sections were washed 3 × 10 min with PBS, mounted on to slides and coverslipped with Flouromount-G mounting medium (Southern Biotech)±DAPI (Roche, 0.5 μg ml^−1^). Images were acquired using widefield epifluorescence (Olympus BX53 or Zeiss AxioObserver) or confocal microscopes (Zeiss LSM 780 or Leica SP5).

For the colocalization of TH with mCherry, images of VTA were acquired using a 20 × objective (Hamamatsu NanoZoomer) with identical acquisition settings across slides. Coronal sections were identified aligning to Paxinos & Watson Bregma points (−3.1, −3.4 and −3.8) from each of four mice. Counting was performed manually using NDP viewer software (Hamamatsu, Japan) to quantify mCherry^+^ VTA cells with clearly labelled soma, and then scored for co-labeling with TH.

For the quantification of c-Fos, mice were tested in a nosepoke discrimination task for 30 min (see below) and perfused 90 min after the beginning of the task. Immunohistochemistry was performed for c-Fos and TH. Images were acquired using a 10 × objective (Olympus BX53) with identical acquisition settings across slides (*n*=4 controls; 4 ChR2 mice) and aligned to reference atlas images (in mm relative to Bregma): VTA (−3.1, −3.4 and −3.8). ImageJ was used to manually count the number of c-Fos positive neurons by an experimenter blind to treatment groups.

To count the number of cells containing retrobeads, coronal images were acquired using a 10 × objective (Zeiss AxioObserver) at the injection site and throughout the VTA; 9 VTA sections per animal between −2.9 to −3.9 mm relative to Bregma, VP/NAc *n*=4; VP/LHb *n*=2; LHb/NAc *n*=1. Zen software (Zeiss) was used to count cells containing red-, green- or both-coloured beads.

### 2-nosepoke discrimination task

Mice were fed *ad libitum* or placed on a restricted feeding schedule as specified. Food restriction consisted of removing food the evening before the first day and access was then restricted to a 3-h period following the assay. At the beginning of the session, ferrules were connected to a 50-μm optical patch cable connected to an optical commutator (Doric Lenses, Canada) and mice were placed in operant chambers (Med Associates) controlled by MedPC IV software. The start of the session was signalled by a brief tone (2 kHz, 0.5 s), illumination of overhead house light, and LED cue lights over the nosepoke holes; sessions were 60 min unless specified. The chamber contained two photobeam-equipped nosepoke holes which were each baited at the start of each session with a sucrose pellet (Bio-Serv, F0071). Beam-breaks on the active nosepoke led to a 0.5 s tone, the LED cue lights over the nosepokes turned off for the duration of the photostimulus unless specified, and the activation of a TTL-controlled DPSS laser (473 nm, Shanghai or OEM laser) set to deliver pulses at 10 mW (80 mW mm^−2^ at 200-μ fibre tip) at 20 or 40 Hz (1–20 s) with a 10-ms pulse width controlled by a Master-8 (A.M.P.I.) or customized Arduino stimulus generator. The output of the laser power was measured using a digital power meter (Thorlabs PM100D/S121C). Nosepokes that occurred during ongoing photostimulation were recorded but without effect; inactive nosepokes led to identical tone and cue light effects but did not trigger the laser.

For behavioural pharmacology studies, naïve mice were exposed to the task until their responding appeared stable over 3 days. Over the subsequent 2 days mice were injected with either vehicle or with a combination of SCH23390 (Tocris, 50 μg kg^−1^ i.p.) and sulpiride (Tocris, 50 mg kg^−1^ i.p.) 30 min before beginning of experiment and using a counter-balanced design.

### 5-choice nosepoke instrumental task

Mice were tethered to the patch cable as described for the 2-nosepoke discrimination task. Identical chambers, lasers and conditions were used but a 5-nosepoke wall (Med Associates) was used in place of the two nosepoke holes. Each of 5-nosepoke holes led to stimulation that varied by pulse number (that is, duration) or frequency as specified, sessions were 45 min unless specified, and all other parameters were as described in the nosepoke discrimination task.

### Two-bottle choice task

Before the first day of testing mice were water-deprived overnight and subsequently provided restricted access for 3 h daily at the end of each session. On day 1 (baseline) mice were provided water through two identical sippers and licks were recorded for 45 min using contact lickometers (Med Associates). On subsequent days one sipper was designated ‘active' and the active sipper was assigned in a balanced manner such that on average no preference was present on baseline day. Every fifth lick on the active sipper led to a photostimulation of 40 pulses at 40 Hz (1 s) with a 10 ms pulse width at 10 mW. Licks on the inactive sipper were without effect. Licks during the photostimulation were recorded but did not contribute to triggering the next photostimulation; mice generally discontinued licking on photostimulation and often resumed shortly thereafter. On days 5–8 the inactive sipper was filled with escalating concentrations of sucrose. For behavioural pharmacology studies mice were injected with either SCH23390 (Tocris, i.p.) or sulpiride (Tocris, i.p.) 30 min before beginning of experiment. The total number of licks on the active and inactive side of sipper and the total number of photostimulations were recorded via Med-PC IV. Preference was calculated using active licks divided by the sum of all licks; on five occasions mice made zero licks on one of the two sippers (4 controls and 1 ChR2) and were excluded from preference calculations.

### Real-time place procedure

On a baseline (pre-test) day mice were placed on the border between two adjoining compartments (20 × 20 cm) and the amount of time spent in each compartment was recorded using video tracking software (Anymaze). Most mice displayed no preference, but those with greater than 80% side preference on pre-test were excluded from further study. On subsequent days one side was designated active and entry to the active side triggered photostimulation (0–40 Hz, 5–10-ms pulse width, 10 mW), using the lasers as described above but controlled by an ANY-maze interface (San Diego Instruments). In some sessions a post-test day was included that was identical to the pre-test day. Sessions lasted for 25 min unless specified and the amount of time spent in each compartment and number of crossings was recorded. The Halorhodopsin experiment was performed identically except for the photostimulation (continuous 10 mW, 532 nm, Shanghai laser) and the duration of each session (30 min).

### Electrophysiological recordings from adult brain slices

Adult mice (7–12 weeks) were deeply anaesthetized with pentobarbital (200 mg kg^−1^ i.p.; Virbac) and perfused intracardially with 10 ml ice-cold sucrose-artificial cerebrospinal fluid (ACSF) containing (in mM): 75 sucrose, 87 NaCl, 2.5 KCl, 7 MgCl_2_, 0.5 CaCl_2_, 1.25 NaH_2_PO_4_, 25 NaHCO_3_ and continuously bubbled with carbogen (95% O_2_–5% CO_2_). Brains were extracted and 200-μm coronal slices were cut in sucrose-ACSF using a Leica Vibratome (vt1200). Slices were transferred to a perfusion chamber containing ACSF at 31 °C (in mM): 126 NaCl, 2.5 KCl, 1.2 MgCl_2_, 2.4 CaCl_2_, 1.4 NaH_2_PO_4_, 25 NaHCO_3_, 11 glucose, continuously bubbled in carbogen. After at least 45 min recovery, slices were transferred to a recording chamber continuously perfused with ACSF (2–3 ml min^−1^) maintained at 29–31 °C using an in-line heater. Patch pipettes (3.5–5.5 MΩ) were pulled from borosilicate glass (King Precision Glass) and for voltage-clamp recordings filled with internal recording solution containing (in mM): 120 CsCH_3_SO_3_, 20 HEPES, 0.4 EGTA, 2.8 NaCl, 5 TEA, 2.5 Mg-ATP, 0.25 Na-GTP, at pH 7.25 and 285±5 mOsm. For current-clamp and cell-attached recordings potassium-based recording solution was used (in mM): 123 CH_3_KO_3_S, 10 HEPES, 0.2 EGTA, 8 NaCl, 2.5 Mg-ATP, 0.25 Na-GTP, at pH 7.25 and 280±5 mOsm.

mCherry-labelled VGLUT2^+^ VTA neurons and terminals were visualized by epifluorescence and visually guided patch recordings were made using infrared-differential interference contrast (IR-DIC) illumination (Axiocam MRm, Examiner.A1, Zeiss). ChR2 was activated by flashing blue light (5-ms pulse width to induce postsynaptic currents in whole-cell configuration and 1-ms pulse width in 40-Hz trains for 5 s in cell-attached recordings) through the light path of the microscope using a light-emitting diode (UHP-LED460, Prizmatix) under computer control. Excitatory and inhibitory postsynaptic currents (E/IPSCs) were recorded in whole-cell voltage clamp and action potentials were recorded in whole-cell current clamp (*I*=0) or cell-attached modes (Multiclamp 700B amplifier, Axon Instruments), filtered at 2 KHz, digitized at 10 KHz (Axon Digidata 1550, Axon Instruments), and collected online using pClamp 10 software (Molecular Device). Series resistance and capacitance were electronically compensated before recordings. Estimated liquid-junction potential was 12 mV and left uncorrected. Series resistance and/or leak current were monitored during recordings and cells that showed >25% change during recordings were considered unstable and discarded.

Neurons were held in voltage-clamp at −60 mV to record AMPAR EPSCs and at 0 mV to record GABA_A_R IPSCs in whole-cell configuration. For whole-cell voltage-clamp recordings, single-pulse (5-ms) photostimuli were applied every 55 s and 10 photo-evoked currents were averaged per neuron per condition. For cell-attached or current-clamp studies of spike fidelity and cell-attached studies on firing rate, photostimuli trains were delivered every 30 s and three responses averaged per neuron. Action potential frequency was averaged over the 5 s before, during and after the 5-s stimulation train. DMSO or H_2_O stock solutions of drugs were diluted 1,000-fold in ACSF and bath applied at the following concentrations: 6,7-dinitroquinoxaline-2,3-dione (DNQX, 10 μM, Sigma), picrotoxin (10 μM, Sigma), gabazine (10 μM, Tocris), tetrodotoxin (1 μM, Tocris) and 4-aminopyridine (500 mM, Tocris). Current sizes were calculated by using peak amplitude from baseline. Decay time constants (*τ*) were calculated by fitting an exponential function to each averaged current trace using the following formula: f(t)=e^–t/*τ*^+C.

### Statistics

To evaluate statistical significance, data were subjected to Student's *t* test or ANOVA, in some experiments followed by *post hoc* analysis (KyPlot, Prism or Statistica) as described in Supplementary Tables 1 and 2. Statistical significance was set at *P*≤0.05. All data are presented as means±s.e.m. unless noted.

### Data availability

Data are available from the corresponding author (T.S.H.) upon request.

## Additional information

**How to cite this article:** Yoo, J. H. *et al*. Ventral tegmental area glutamate neurons co-release GABA and promote positive reinforcement. *Nat. Commun.* **7,** 13697 doi: 10.1038/ncomms13697 (2016).

**Publisher's note:** Springer Nature remains neutral with regard to jurisdictional claims in published maps and institutional affiliations.

## Supplementary Material

Supplementary Information Supplementary Figures and Supplementary Tables.

Supplementary Movie 1 Mouse nosepokes for optogenetic self-stimulation (40 Hz, 80 pulses) of VGLUT2+ VTA neurons.

Supplementary Movie 2 Mouse in a real-time place procedure making multiple brief entries into a compartment paired with sustained 40 Hz optogenetic stimulation of VGLUT2+ VTA neurons.

## Figures and Tables

**Table 1:**
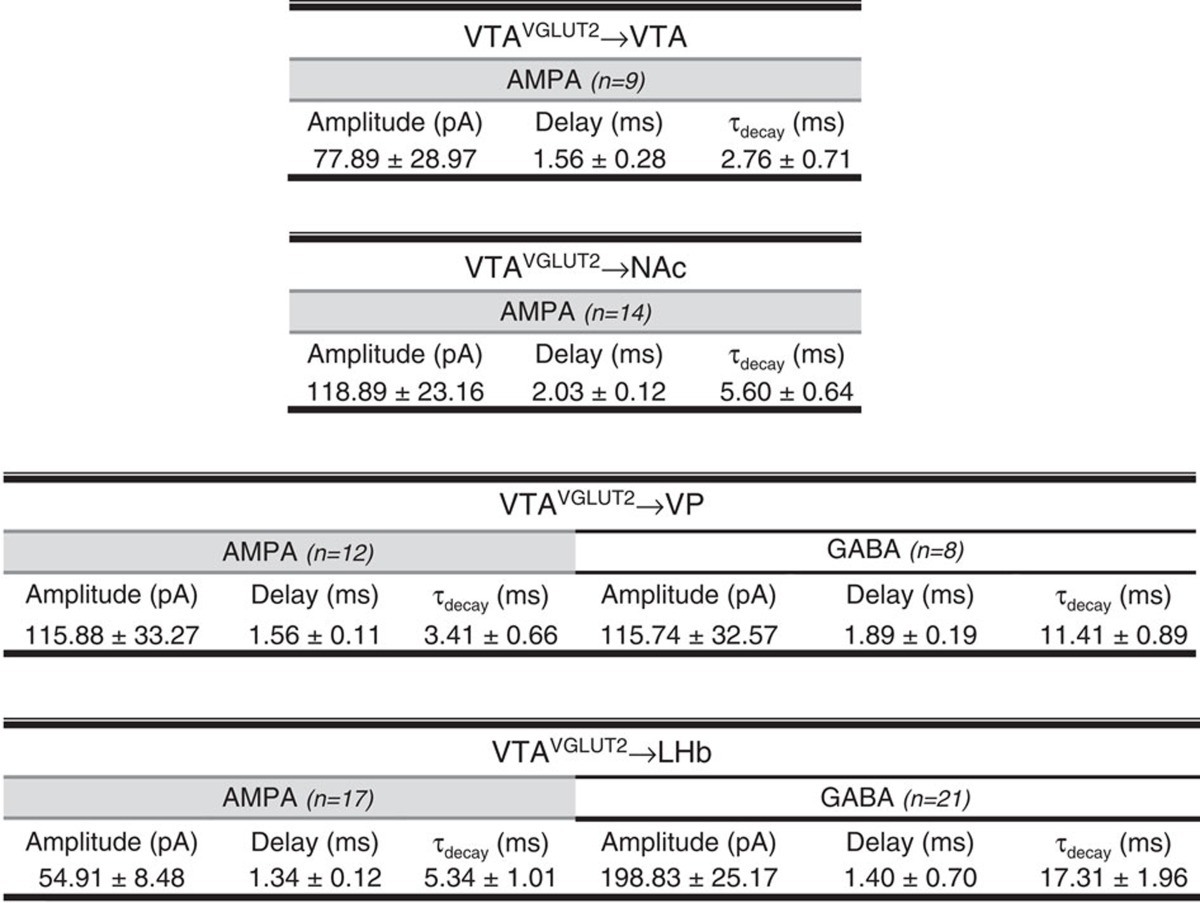
Properties of light-evoked excitatory and inhibitory synaptic currents.
