# Supplementary material for: Ventral tegmental area glutamate neurons co-release GABA and promote positive reinforcement
Source: Nat Commun. 2016 Dec 15;7:13697. doi: 10.1038/ncomms13697 (PMC5171775; doi:10.1038/ncomms13697)
Supplement: Supplementary Information — Supplementary Figures and Supplementary Tables. [file ncomms13697-s1.pdf]

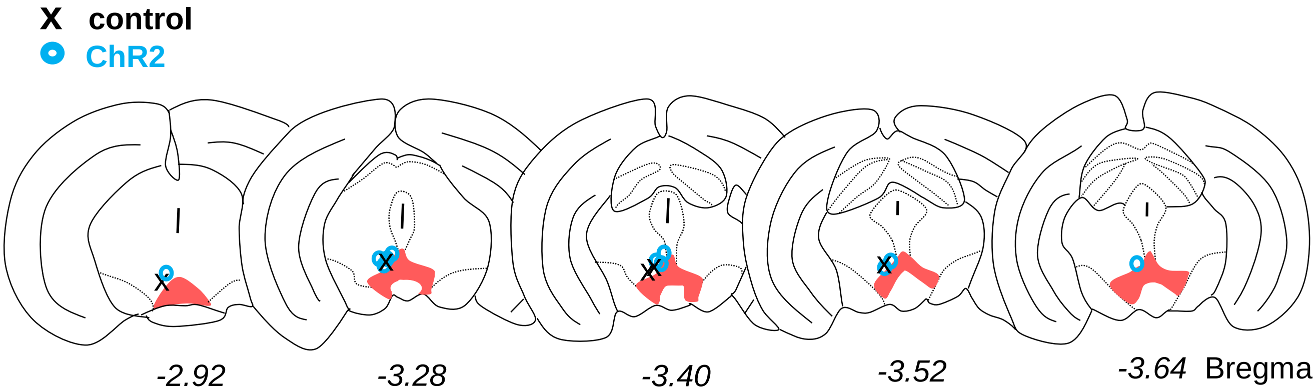

Supplementary Figure 1. ***Placement of optic fibers in VTA.*** Anatomical location of implanted optic fibers identified around the VTA.

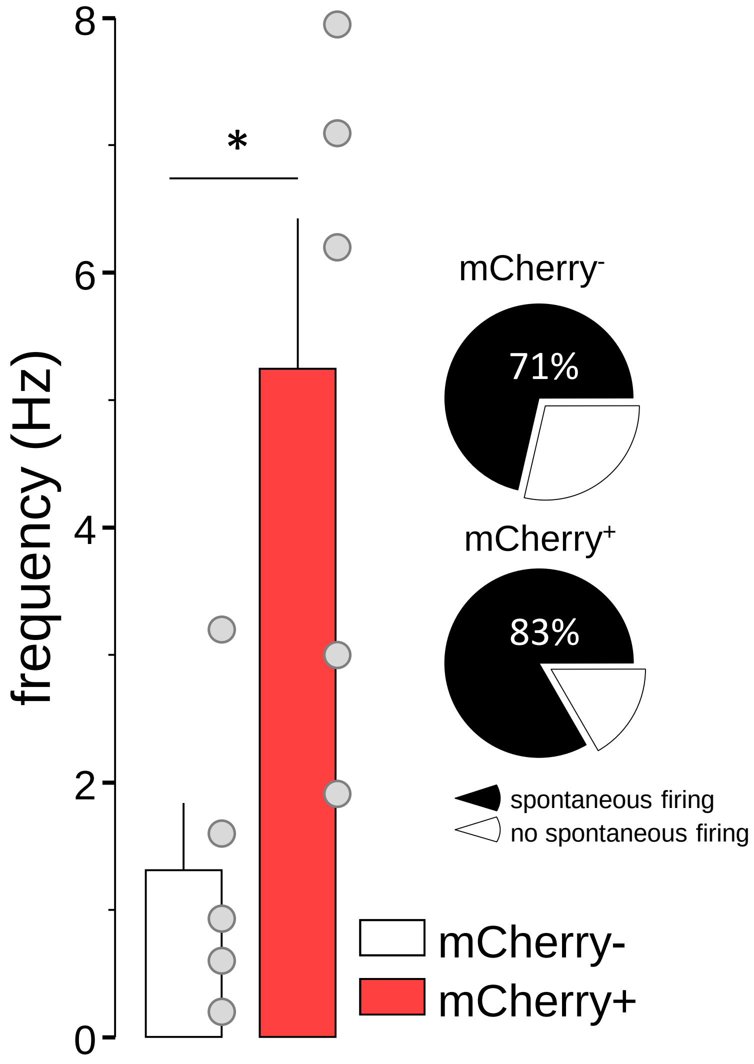

Supplementary Figure 2. ***Spontaneous firing rates of VGLUT2⁺ and mCherry-negative VTA neurons.*** VGLUT2⁺ neurons were identified on the basis of their mCherry expression (mCherry⁺) and showed a significantly higher spontaneous firing frequency (cell-attached) than neighboring mCherry-negative neurons also in the VTA; gray circles represent individual values, *p<0.05. Pie chart insets show the fraction of spontaneously firing neurons in each group.

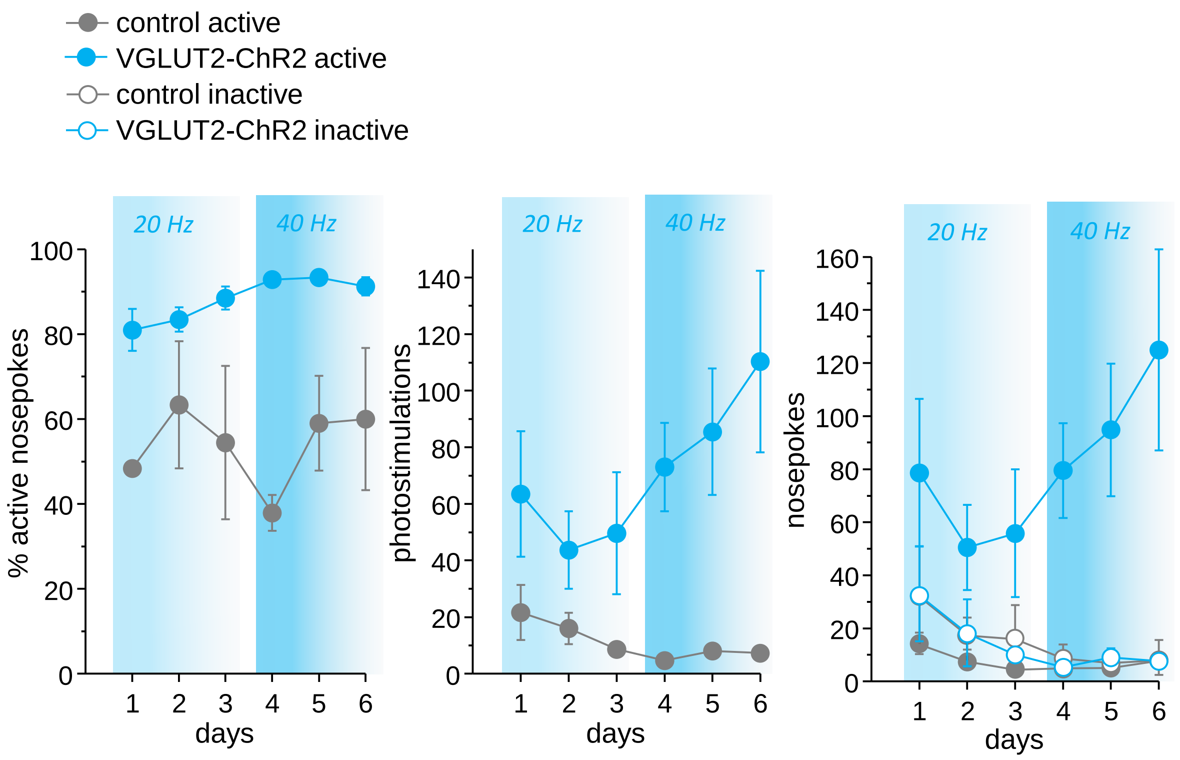

Supplementary Figure 3. ***Photostimulation of VGLUT2⁺ VTA neurons using alternate VGLUT2-Cre line is reinforcing in operant task.*** BAC transgenic VGLUT2-Cre mice expressing ChR2 develop a strong preference for the laser-coupled side compared to Cre-negative littermate controls in the 2-nosepoke discrimination task. ChR2-expressing mice make more active nosepokes to trigger more photostimuli than control mice; $p<0.01$.

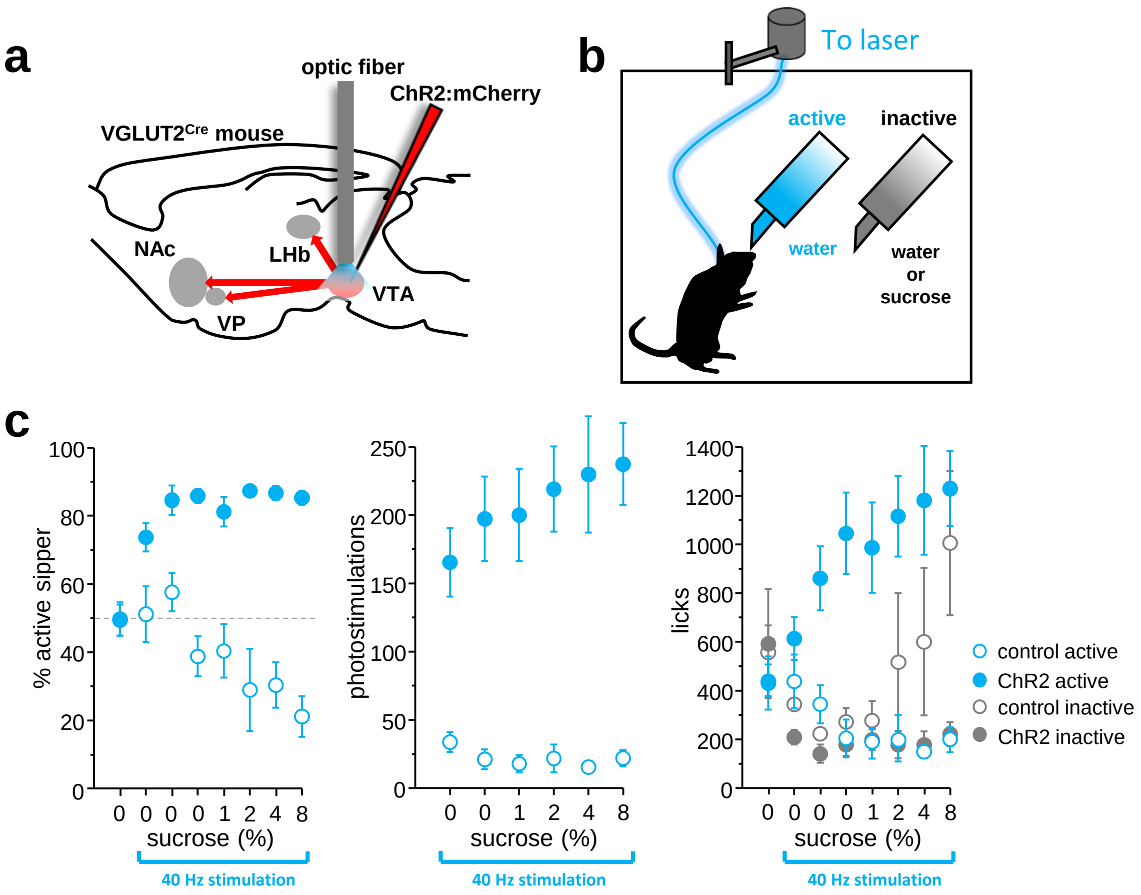

Supplementary Figure 4. ***Photostimulation of VGLUT2$^+$ VTA neurons is reinforcing in 2-bottle choice assay.*** (**a**) Schematic illustrating strategy for selective expression and photostimulation of ChR2 in VGLUT2$^+$ VTA neurons. (**b**) Schematic illustrating 2-bottle choice task where five licks on the active sipper (contains water) trigger 40 pulses at 40 Hz and the inactive sipper contains either water or escalating concentrations of sucrose. (**c**) ChR2-expressing mice but not control mice develop a preference for active sipper; $p<0.001$. This preference is resistant to competition against sucrose, while control mice show no initial preference but develop a preference for the inactive sipper when water is replaced with escalating concentrations of sucrose. ChR2-expressing mice trigger more photostimuli; $p<0.001$ and make more licks on the active sipper than control littermates; $p<0.05$.

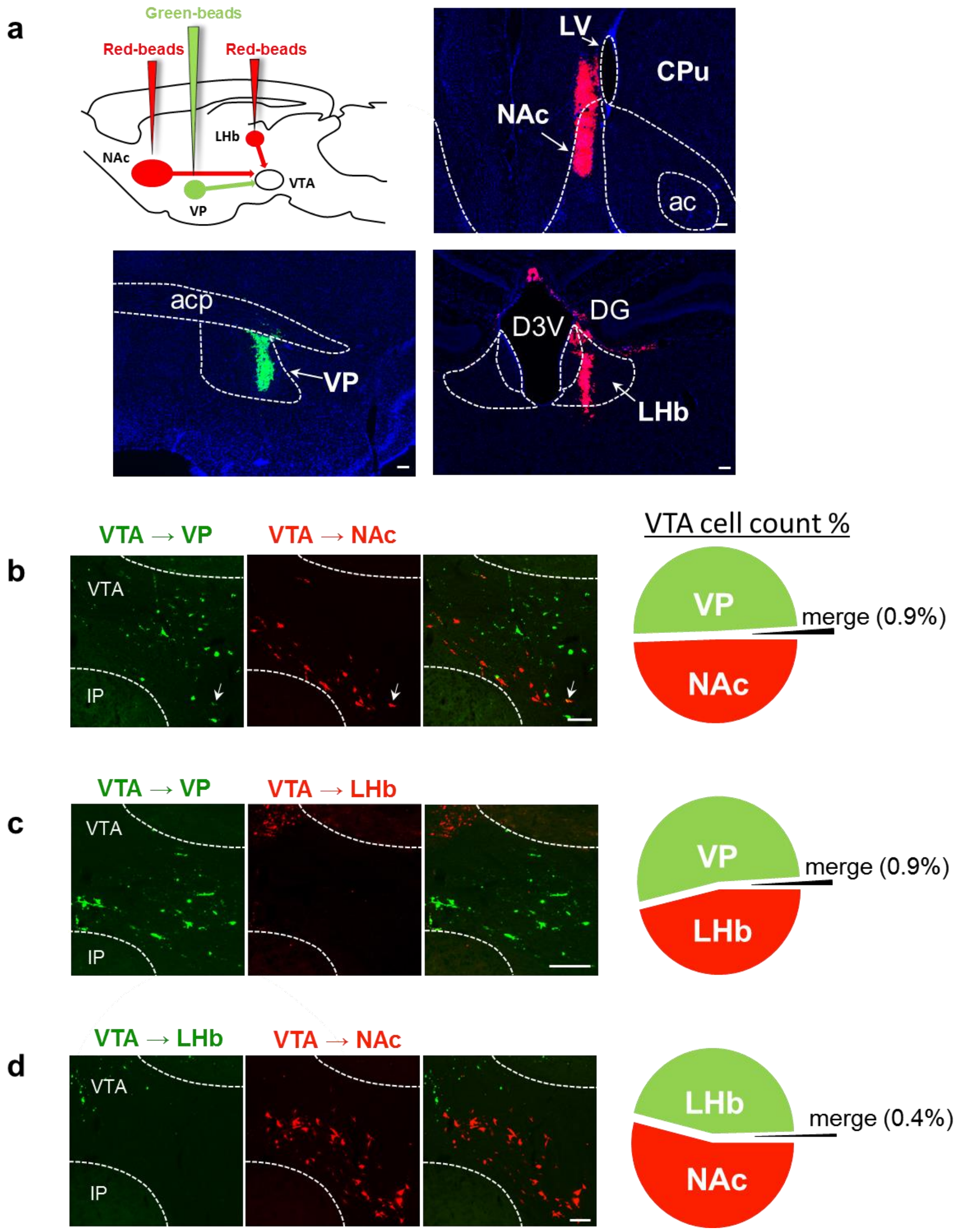

Supplementary Figure 5. Separate populations of VTA neurons target NAc, VP, or LHb. (a) Mice received two unilateral injections on the same side into the VP/NAc-medial shell, VP/LHb or LHb/NAc-medial shell with two different color RetroBeads (red and green). Images of RetroBead injection sites in the NAc-medial shell, VP or LHb; note that NAc targets included some spread to frontal cortex. **(b)** Pie chart of VTA neurons containing retrogradely labeled beads projecting to VP (green; 814 cells; n=4 mice) or NAc (red; 816 cells; n=4 mice), fewer than 1% of cells contained both colored beads. Arrow indicates double labeled cell. **(c)** Pie chart of VTA neurons containing retrogradely labeled beads projecting to VP (green; 342 cells; n=2 mice) or LHb (red; 300 cells; n=2 mice), fewer than 1% of cells contained both colored beads. **(d)** Pie chart of VTA neurons containing retrogradely labeled beads projecting to LHb (green; 255 cells; n=1 mice) or NAc (red; 301 cells; n=1 mice), fewer than 1% of cells contained both colored beads. Scales, 100µm; LV, lateral ventricle; NAc, nucleus accumbens; CPu, caudate putamen; ac/acp, anterior commissure; VP ventral pallidum; DG dentate gyrus; LHb Lateral Haenula; D3V dorsal third ventricle; VTA ventral tegmental area.

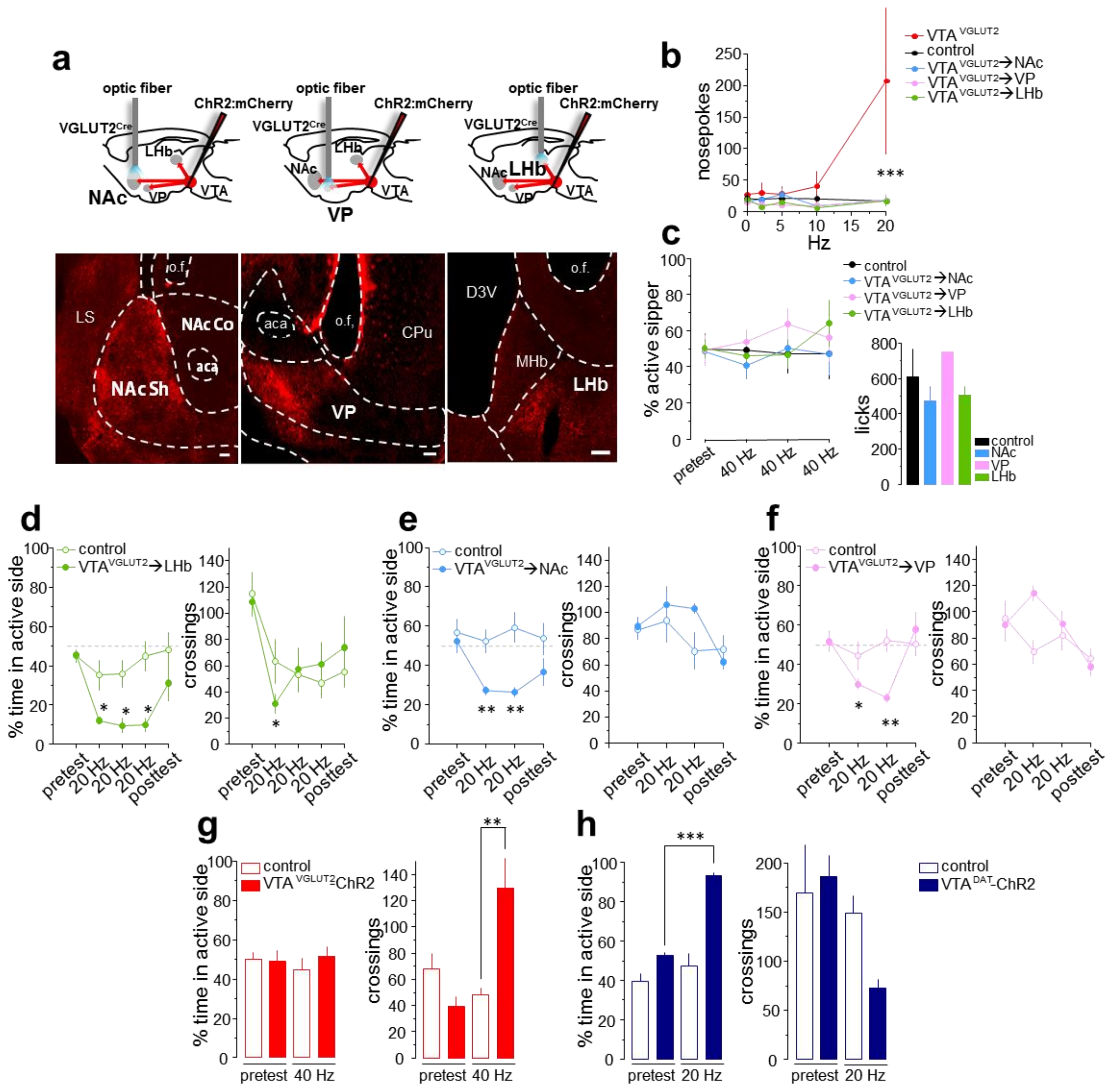

Supplementary Figure 6. ***Photostimulation of VGLUT2⁺ VTA terminals can induce apparent avoidance by RTPP, but not necessarily aversion.*** **(a)** Schematics illustrating strategy for targeting VGLUT2⁺ VTA terminals for photostimulation and example coronal sections with ChR2:mCherry labeling (red); scale, 100mm. (**b**) Using the 5-nosepoke assay coupled to different rates of stimulation (0 to 20 Hz, 1 sec), only stimulation of VGLUT2⁺ cell bodies shows a strong preference; $p<0.001$. (**c**) In the two-bottle choice assay, mice fail to develop a preference or avoidance for an active sipper coupled to NAc, VP, or LHb terminal photostimulation (40 pulses at 40Hz for every 5th lick). (**d**) On a real-time place procedure(RTPP) , ChR2-expressing mice spend significantly less time in the chamber coupled to bilateral optical stimulation of VGLUT2⁺ VTA terminals in the NAc (**e**) or VP (**f**), or unilateral stimulation of terminals in the LHb relative to pre-test (left); but show a decrease in the number of crossings between compartments compared to controls (right); *$p<0.05$, **$p<0.01$. (**g**) On a RTPP test, ChR2-expressing mice do not display a preference or avoidance for the chamber coupled to unilateral optical stimulation of VGLUT2⁺ VTA cell bodies relative to pre-test (left); however, they do increase the number of crossings between compartments (right); **$p<0.01$. (**h**) On a RTPP test, ChR2-expressing mice display a preference for the chamber coupled to unilateral optical stimulation of DAT⁺ VTA cell bodies relative to pre-test (left); ***$p<0.001$.

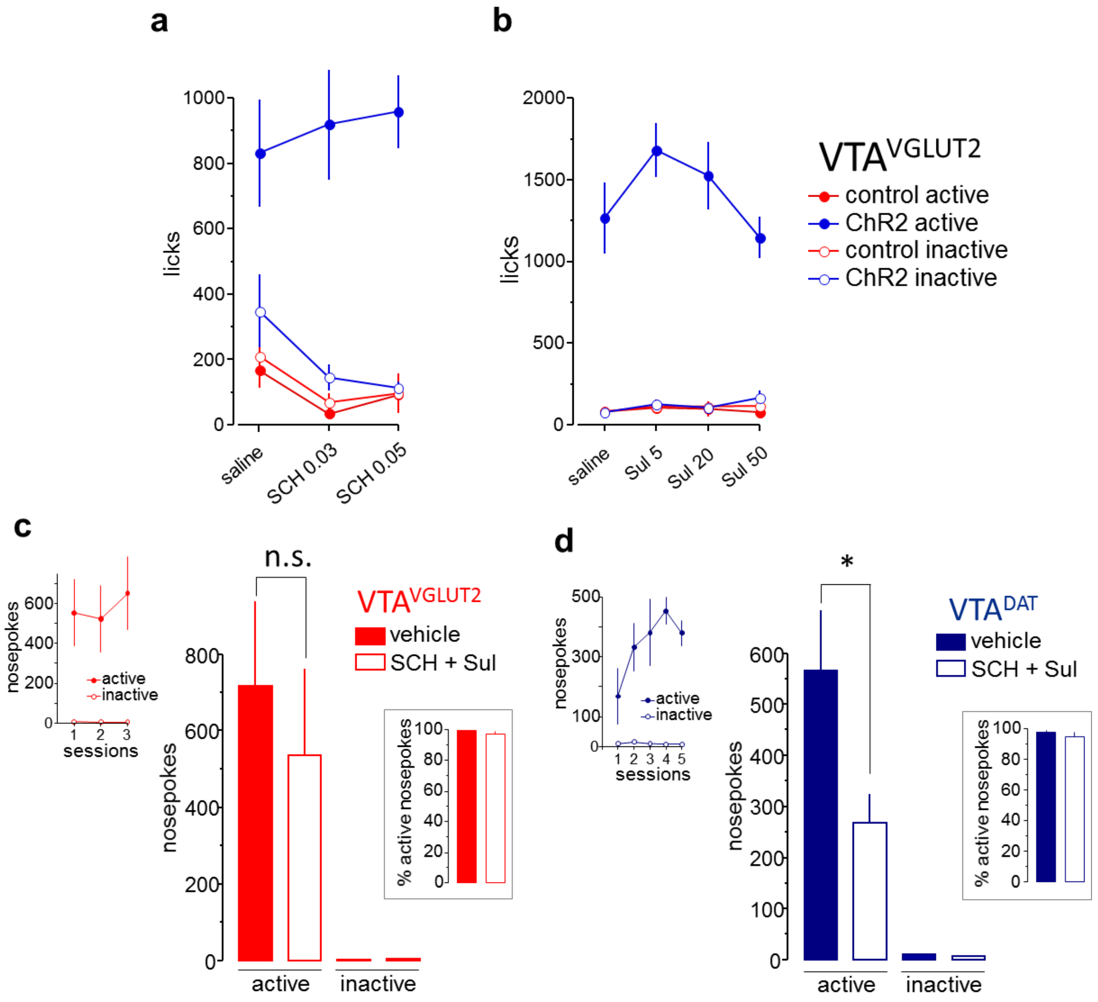

Supplementary Figure 7. ***Operant responding for VGLUT2+ VTA neuron stimulation is resistant to inhibition by dopamine receptor antagonists.*** Using a 2-bottle choice assay, water-restricted mice expressing ChR2 in VGLUT2+ VTA neurons made more licks on an active sipper (dispensing water) compared to an inactive sipper (also dispensing water), or compared to control mice; $p>0.01$. (**a**) Pre-treatment (i.p.) with D_1R or (**b**) D_2R antagonists failed to blunt licking on the active sipper. (**c**) Similar results were obtained with a 60-min 2-nosepoke instrumental assay. Mice were exposed to the assay until they showed stable responding for three consecutive days (inset) and then subjected to combined treatment with 0.05 mg/kg SCH23390 and 50 mg/kg sulpiride (i.p.) or vehicle in a counter-balanced fashion; antagonists did not significantly alter preference (small inset) or the number of nosepokes. (**d**) In contrast, when ChR2 was targeted to DAT+ VTA neurons, the same antagonist treatment significantly attenuated the number of nosepoke responses $*p<0.05$.

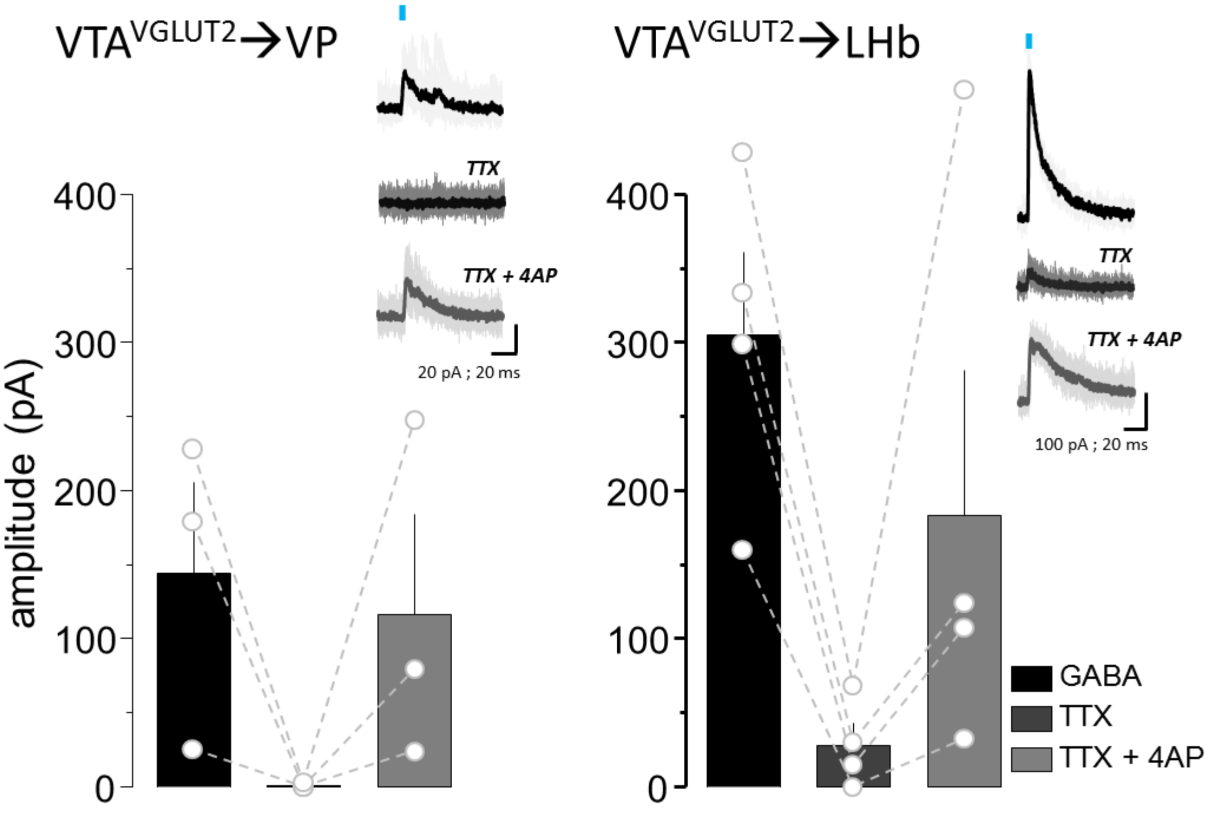

Supplementary Figure 8. ***GABA release from VGLUT2⁺ VTA terminals in VP and LHb is monosynaptic.*** IPSCs recorded at 0 mV were blocked by TTX indicating that light-evoked IPSCs were initially dependent on presynaptic action potential propagation driven by ChR2-mediated activation of voltage-gated sodium channels; but recovered following bath application of the voltage-dependent potassium channel blocker 4-AP (to enhance the ability of ChR2-mediated currents to directly trigger synaptic release). Bar's represent means, grey circles and dashed lines represent responses of individual neurons, and insets show representative traces.

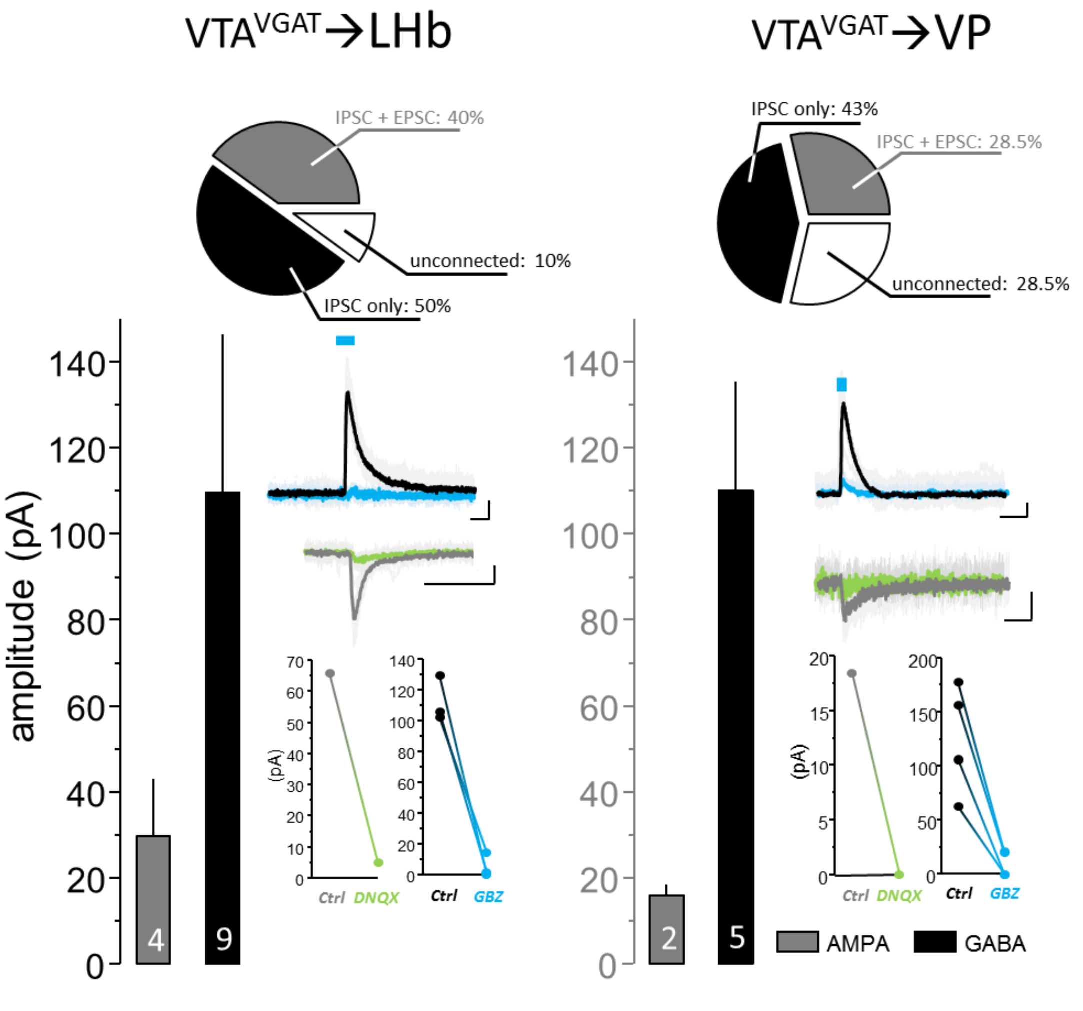

Supplementary Figure 9. ***Glutamate co-release from VGAT⁺ VTA terminals in VP and LHb.*** ChR2 was expressed in VGAT⁺ (GABA) neurons in VTA and terminals in VP and LHb were photostimulated. Pie charts show the percentage of recorded neurons with specified light-triggered responses in the LHb (left) or VP (right). Bars show mean amplitudes with average amplitudes of light-triggered EPSCs recorded at -60 mV and IPSCs recorded at 0 mV; white numbers in bar correspond to number of neurons. Top insets show representative traces of DNQX-sensitive AMPA EPSCs (grey/light green) and GBZ-sensitive GABA IPSCs (black/light blue). Bottom insets show individual photo-responses ± DNQX (10 µM) on light-triggered EPSC amplitude and ± gabazine (GBZ, 10 µM) on IPSC amplitude. Scales: 50 pA, 20 ms.

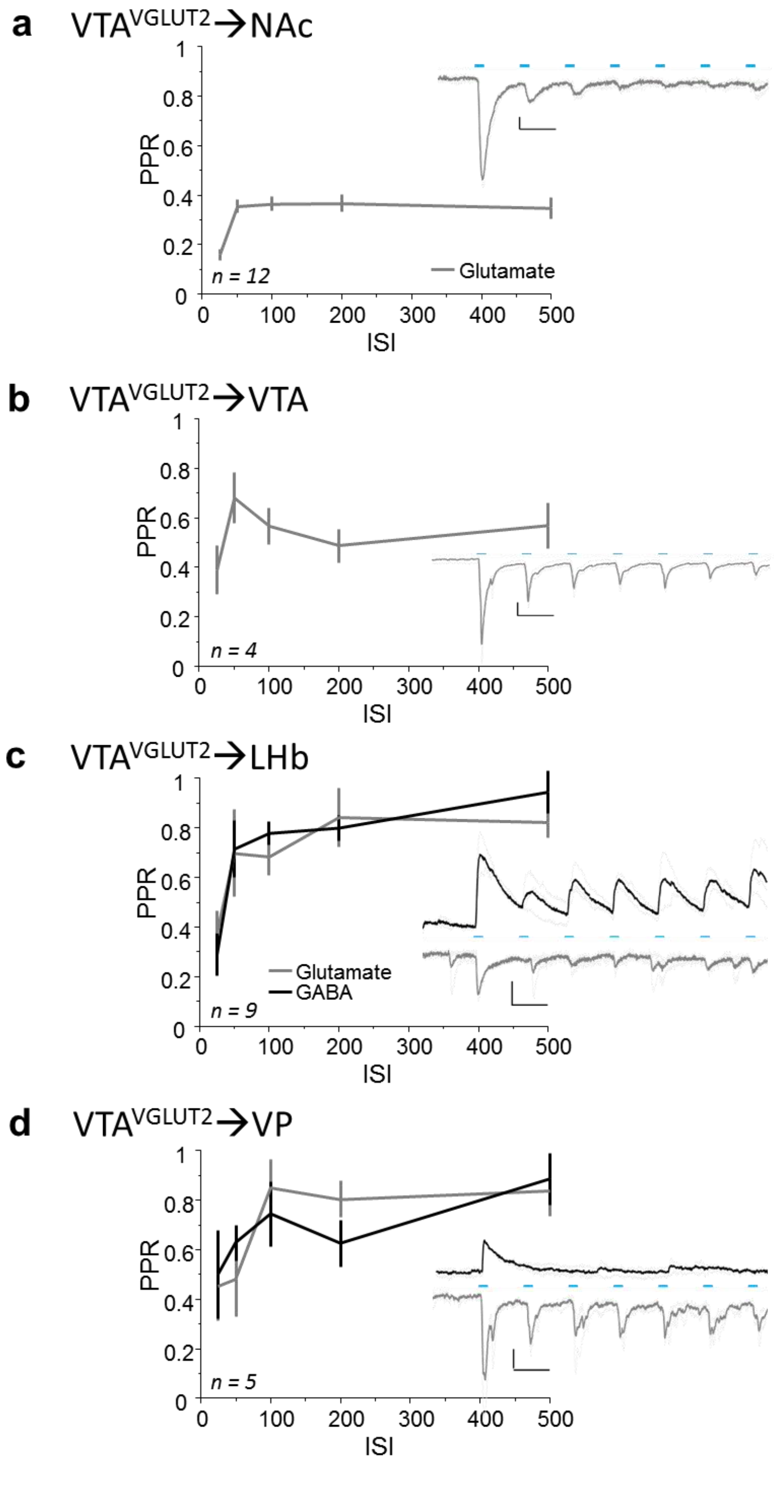

Supplementary Figure 10. ***Massive frequency-dependent synaptic depression at VGLUT2⁺ VTA synapses.*** Terminal photostimulation using different frequencies produced a strong and sustained depression of light-evoked EPSCs in (**a**) NAc, (**b**) VTA, and both EPSCs and IPSCs in (**c**) LHB and (**d**) VP. Plots represent paired-pulse ratios (PPR) at different inter-stimulus intervals (ISI). Insets shows representative traces of 40-Hz evoked EPSCs recorded at V_h= -60 mV (grey traces) and IPSCs recorded at V_h= 0 mV (black traces). Scales: 20 pA, 20 ms.

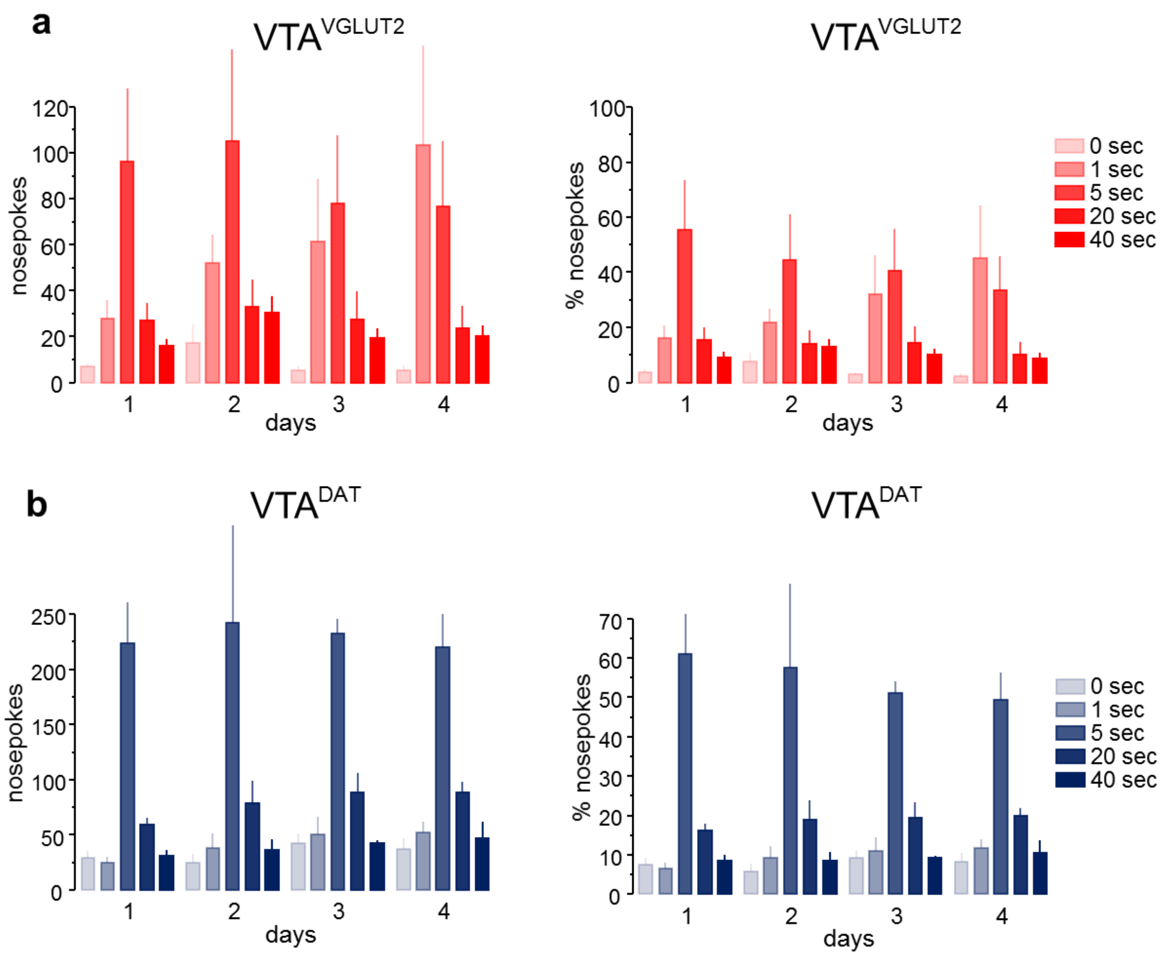

Supplementary Figure 11. ***All four days of stimulation in the 5-nosepoke assay with varied stimulus duration.*** (**a**) Using a 5-nosepoke choice assay with 40-Hz stimulation coupled to different stimulus durations, photostimulation of VGLUT2^{+} VTA neurons produces a descending preference for shorter (≤ 5s) stimulus durations that becomes more pronounced with repeated exposure to the task. Using the same task, (**b**) photostimulation of DAT^{+} VTA neurons shows response distributions shifted toward relatively longer durations (≥ 5s).

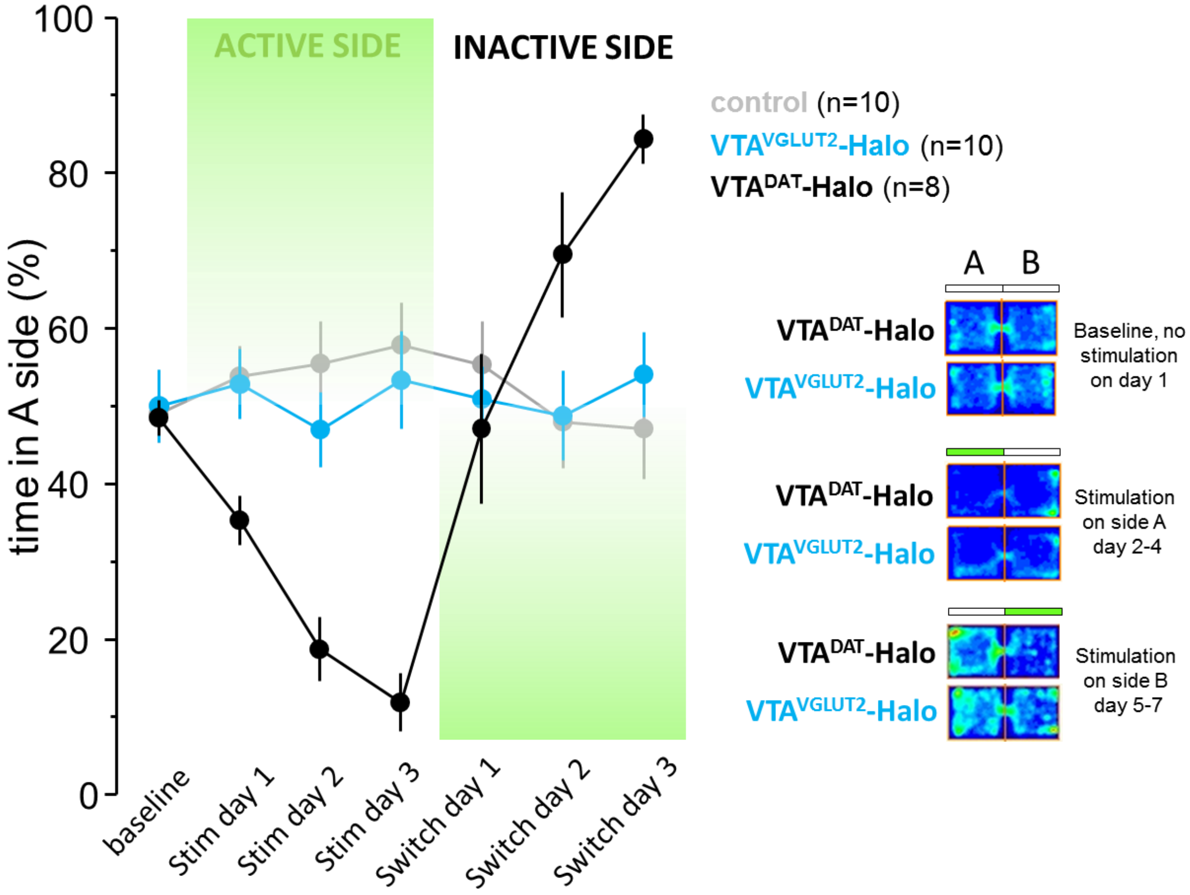

Supplementary Figure 12. Optogenetic inhibition of VTA dopamine but not glutamate neurons produces real time place avoidance. A real time place procedure was performed on DAT-Cre and VGLUT2-Cre mice expressing Halorhodopsin or YFP (control) in VTA. DAT-Cre mice expressing Halo in dopamine neurons showed reduced time spent in the compartment during stim days (2-4) and switched sides when the stim-paired compartment was switched (days 5-7); VGLUT2-Cre mice expressing Halo in glutamate neurons, or YFP-expessing control mice, showed neither preference nor aversion to the side coupled to green laser. Genotype $p < 0.001$; day $p < 0.05$; genotype x day interaction $p < 0.001$.

Figure		Description	N-number	Statistic	P value	Post-hoc	Post-hoc P value
1	A	schemetic					
	B	mCherry+	738 cells, 4 slices, 4 mice (-3.1 mm)<br>436 cells, 4 slices, 4 mice (-3.4 mm)<br>356 cells, 4 slices, 4 mice (-3.8 mm)				
		mCherry+/TH+	48 cells, 4 slices, 4 mice (-3.1 mm)<br>61 cells, 4 slices, 4 mice (- 3.4 mm)<br>61 cells, 4 slices, 4 mice (- 3.8 mm)				
	1B inset	mCherry+/TH+	4 mice				
	C	sample image					
	D	c-Fos+ control	997 cells, 4 slices, 4 mice				
		c-Fos+ ChR2:mCherry	2734 cells, 4 slices, 4mice				
		c-Fos+ control	506 cells, 4 slices, 4mice (-3.1 mm)<br>230 cells, 4 slices, 4 mice (-3.4 mm)<br>261 cells, 4 slices, 4 mice (-3.8 mm)	**2-way ANOVA**<br>F (2, 12) = 12.85, bregma<br>F (1, 6) = 30.52, expression	< 0.01<br>< 0.01	**no *post-hoc* conducted (NPC)**	
		sample image					
	E	mCherry+ spike fidelity	10 cells, 6 mice				
2	A<br>B	schemetic					
	C	% active nosepokes	control: 5 mice<br>VG2-ChR2: 11 mice	**repeated measure (RM) 2-way ANOVA**<br>$F_{(1,14)}$ = 53.06, expression<br>$F_{(3,42)}$ = 2.828, day X expression	< 0.001<br>< 0.05	**NPC**	
	D	phostostimulations		**RM 2-way ANOVA**<br>$F_{(1,14)}$ = 18.80, expression<br>$F_{(3,42)}$ = 5.657, day X expression	< 0.001<br>< 0.01		
	E	nosepokes		**RM 3-way ANOVA**<br>$F_{(1,28)}$ = 14.829, expression<br>$F_{(1,28)}$ = 24.799, nosepoke<br>$F_{(1,28)}$ = 18.014, expression X nosepoke	< 0.001<br>< 0.001<br>< 0.001		
	F	schematic					
	G	% active nosepokes	VG2-ChR2: 10 mice	**One-way ANOVA**<br>F(4,45) = 8.142, frequency	< 0.001	**Bonferroni**<br>vs 0 Hz	* < 0.05<br>** < 0.01<br>*** <0.001
	H	photostimulation		One way ANOVA - $F_{(4,45)}$ = 6.155, frequency	< 0.001		
	I	nosepokes		One way ANOVA - $F_{(4,45)}$ = 6.027, frequency	< 0.001		
	J	nosepokes	control: 6 mice	**2-way ANOVA**<br>$F_{(1,40)}$ = 7.6969, nosepoke hole	< 0.01	**Fisher LSD**<br>vs control active	
			VTA^{VGLUT2} → NAc: 6 mice				ns
			VTA^{VGLUT2} → VP: 6 mice				**< 0.01
			VTA^{VGLUT2} → LHb: 6 mice				ns
	K	% nosepokes	control: 6 mice<br>VTA^{VGLUT2} → NAc: 6 mice<br>VTA^{VGLUT2} → VP: 6 mice<br>VTA^{VGLUT2} → LHb: 6 mice	**One-way ANOVA**<br>$F_{(3,20)}$ = 2.95, expression	0.057	**NPC**	
3	A	VTA	AMPA: 9 cells, 4 mice<br>AMPA + DNQX: 2 cells, 1 mouse				
	B	NAc	AMPA: 15 cells, 10 mice<br>AMPA + DNQX: 6 cells, 4 mice	**Paired t-test**	* < 0.05<br>** < 0.01<br>*** <0.001	**NPC**	
	C	VP	AMPA: 12 cells, 9 mice<br>AMPA + DNQX: 6 cells, 5 mice	**Paired t-test**			
			GABA: 8 cells, 7 mice<br>GABA + GBZ: 3 cells, 3 mice				
	D	LHb	AMPA: 17 cells, 12 mice<br>AMPA + DNQX: 5 cells. 4 mice	**Paired t-test**			
			GABA: 21 cells, 14 mice<br>GABA + GBZ: 10 cells. 7 mice	**Paired t-test**			
4	A	VP GABA/AMPA ratio<br>LHb GABA/AMPA ratio	VP: 7 cells, 6 mice<br>LHb: 17 cells, 12 mice	**Unpaired t-test**	**<0.01	**NPC**	
	B	example traces					
	C	VP cell attached 40Hz	20 cells, 9 mice	**RM one-way ANOVA**<br>$F_{(2,36)}$ = 16.901	<0.001	**Tukey**: pre-stim vs stim<br>**Tukey**: recovery vs stim<br>**Tukey** : recovery vs pre-stim	<0.001<br><0.001<br>ns
		LHb cell attached 40Hz	18 cells, 9 mice	**RM one-way ANOVA**<br>$F_{(2,36)}$ = 28.969	<0.001	**Tukey**: pre-stim vs stim<br>**Tukey**: recovery vs stim<br>**Tukey**: recovery vspre-stim	<0.001<br><0.001<br>ns
	D	VP cell attached 40 Hz	20 cells, 9 mice				
		LHb cell attached 40Hz	20 cells, 10 mice				
5	A	schemetic (heat map)					
	B	%time active	control: 4 mice<br>VTA^{VGLUT2}: 9 mice<br>VTA^{DAT} : 5 mice	**RM 2-way ANOVA**<br>$F_{(2,15)}$ = 27.2329, expression<br>$F_{(7,105)}$ = 2.7464, frequency<br>$F_{(14,105)}$ = 2.7074, expression X frequency	< 0.001<br>< 0.05<br>< 0.01	**NPC**	
	C	crossings		**RM 2-way ANOVA**<br>$F_{(2,15)}$ = 16.963, expression<br>$F_{(14,105)}$ = 2.0922, expression X frequency	< 0.001<br>< 0.05		
	D	schemetic (heat map)					
	E	%time active	VTA^{VGLUT2} → NAc: 5 mice<br>VTA^{VGLUT2} → VP: 6 mice<br>VTA^{VGLUT2} → LHb: 5 mice	**RM 2-way ANOVA**<br>$F_{(7,91)}$ = 8.3853, frequency	< 0.001	**NPC**	
	F	crossings		**RM 2-way ANOVA**<br>$F_{(14,91)}$ = 2.3745, expression X frequency	< 0.01		
	G	active side visit	VTA^{VGLUT2}: 9 mice	**Kolmogorov-Smirnov**<br>vs 0 Hz	< 0.001		
			VTA^{VGLUT2} → NAc: 5 mice		< 0.001		
			VTA^{VGLUT2} → VP: 6 mice		< 0.001		
			VTA^{VGLUT2} → LHb: 5 mice		< 0.001		
6	A	% nosepokes	VTA^{VGLUT2} : 9 mice<br>VTA^{DAT} : 5 mice	**2-way ANOVA**<br>$F_{(4,60)}$ = 10.4718, duration<br>$F_{(4,60)}$ = 5.0897, duration X genotype	< 0.001<br>< 0.01	**NPC**	
	B	nosepokes					
	C	% nosepokes	9 mice	**RM 2-way ANOVA**<br>$F_{(1,8)}$ = 12.19, duration	< 0.01	**NPC**	
		nosepokes		$F_{(1,8)}$ = 9.533, duration	< 0.05		
	D	% nosepokes	5 mice	**RM 2-way ANOVA**<br>$F_{(4,16)}$ = 23.19, duration X day	< 0.001		
		nosepokes		$F_{(4,16)}$ = 22.43, duration X day	< 0.001		

Supplementary Table 1. Statistical procedures used on data represented in main figures.

Supplementary Figure		Description	N-number	Statistic	P value	Post-hoc	Post-hoc P value
1		schemetic					
2		Firing frequency	mCherry⁺: 5 cells, 3 mice<br>mCherry⁻: 5 cells, 3 mice	**Unpaired t-test**	*< 0.05	**no post-hoc conducted (NPC)**	
3		% active nosepokes	control : 3 mice<br>VG2-ChR2 : 6 mice	**RM 2-way ANOVA**<br>$F_{(1,7)}$ = 20.981, expression<br>$F_{(5,35)}$ = 2.77, day<br>$F_{(5,35)}$ = 3.521, day X expression	< 0.01<br>< 0.05<br>< 0.05		
		photostimulations		**RM 2-way ANOVA**<br>$F_{(5,35)}$ = 2.697, day<br>$F_{(5,35)}$ = 4.287, day X expression	< 0.05<br>< 0.01		
		nosepokes		**RM 3-way ANOVA**<br>$F_{(5,70)}$ = 2.829, day<br>$F_{(5,70)}$ = 4.47, day X expression<br>$F_{(5,70)}$ = 2.526, day X nosepoke	< 0.05<br>< 0.01<br>< 0.05		
4	A<br>B	schemetic					
	C	% active sipper	control: 5 mice<br>VG2-ChR2: 7 mice	**RM 2-way ANOVA**<br>$F_{(1,10)}$ = 134.165, expression<br>$F_{(7,70)}$ = 3.424, day<br>$F_{(7,70)}$ = 9.827, day X expression	< 0.001<br>< 0.01<br>< 0.001	**NPC**	
		photostimulations		**RM 2-way ANOVA**<br>$F_{(1,10)}$ = 25.621, expression<br>$F_{(6,60)}$ = 3.687, day X expression	< 0.001<br>< 0.01		
		licks		**RM 3-way ANOVA**<br>$F_{(1,20)}$ = 5.956, sipper<br>$F_{(1,20)}$ = 4.433, expression<br>$F_{(1,20)}$ = 19.857, expression X sipper<br>$F_{(7,140)}$ = 3.366, day<br>$F_{(7,140)}$ = 2.086, day X expression<br>$F_{(7,140)}$ = 3.385, day X sipper<br>$F_{(7,140)}$ = 9.323, day x expression X sipper	< 0.05<br>< 0.05<br>< 0.001<br>< 0.01<br>< 0.05<br>< 0.01<br>< 0.001		
5	A	schemetic and sample imgaes					
	B	VP and NAc	VP (814 cells) and NAc (816 cells): 4 mice				
	C	VP and LHb	VP (342 cells) and LHb (300 cells): 2 mice				
	D	LHb and NAc	LHb (255 cells) and NAc (301 cells): 1 mouse				
6	A	schemetic and sample imgaes					
	B	nosepokes	control : 6 mice<br>VTA^{VGLUT2} : 5 mice<br>VTA^{VGLUT2} → NAc: 6 mice<br>VTA^{VGLUT2} → VP: 6 mice<br>VTA^{VGLUT2} → LHb: 8 mice	**2-way ANOVA**<br>$F_{(4,96)}$ = 3.266, frequency<br>$F_{(4,24)}$ = 4.209, expression<br>$F_{(16,96)}$ = 2.674, frequency X expression	< 0.05<br>< 0.05<br>< 0.05	**Bonferroni**	
	C	% active sipper	control : 6 mice<br>VTA^{VGLUT2} → NAc: 6 mice<br>VTA^{VGLUT2} → VP: 6 mice<br>VTA^{VGLUT2} → LHb : 6 mice	**2-way ANOVA**<br>$F_{(3,57)}$ = 0.5226, day<br>$F_{(3,19)}$ = 0.4153, expression<br>$F_{(9,57)}$ = 0.5739, day X expression	ns<br>ns<br>ns	**NPC**	
		licks		**One way ANOVA**	ns		
	D	% time in active side	control: 7 mice<br>VTA^{VGLUT2} → LHb: 5 mice	**RM 2-way ANOVA**<br>$F_{(1,10)}$ = 23.646, expression<br>$F_{(4,40)}$ = 4.690, day	< 0.001<br>< 0.01	**Tukey**<br>vs pretest	
		crossings		$F_{(4,40)}$ =13.21, day	< 0.001	vs control	
	E	% time in active side	control: 6 mice<br>VTA^{VGLUT2} → NAc: 5 mice	**RM 2-way ANOVA**<br>$F_{(1,9)}$ = 6.482, expression<br>$F_{(3,27)}$ = 5.986, day<br>$F_{(3,27)}$ = 5.283, expression X day	< 0.05<br>< 0.01<br>< 0.01	**Tukey**<br>vs pretest	* < 0.05<br>** < 0.01<br>*** <0.001
		crossings		$F_{(3,27)}$ = 5.6965, day	< 0.01	vs control	
	F	% time in active side	control: 10 mice<br>VTA^{VGLUT2} → VP: 7 mice	**RM 2-way ANOVA**<br>$F_{(3,45)}$ = 9.521, day<br>$F_{(3,45)}$ = 7.866, expression X day	< 0.001<br>< 0.001	**Tukey**<br>vs pretest	
		crossings		F(3,45) =5.913, day<br>$F_{(3,45)}$ = 3.726, expression X day	< 0.01<br>< 0.05	vs control	
	G	% time in active side	control: 8 mice<br>VTA^{VGLUT2}-ChR2: 8 mice	$F_{(1,14)}$ =2.831, expression X day	ns	**NPC**	
		crossings		**RM 2-way ANOVA**<br>$F_{(1,14)}$ = 6.715, day<br>$F_{(1,14)}$ = 16.476, expression X day	< 0.05<br>< 0.01	**Tukey**<br>vs control	
	H	% time in active side	control: 4 mice<br>VTA^{DAT}-ChR2: 3 mice	**RM 2-way ANOVA**<br>$F_{(1,5)}$ = 24.992, expression<br>$F_{(1,5)}$ = 101.869, day<br>$F_{(1,5)}$ = 45.326, expression X day	< 0.01<br>< 0.001<br>< 0.01	**Tukey**<br>vs pretest	
		crossing		$F_{(1,5)}$ = 8.734, day	< 0.05	**NPC**	
7	A	licks (SCH23390)	control: 4 mice<br>VTA^{VGLUT2} : 10 mice	**RM 3-way ANOVA**<br>$F_{(1,24)}$ = 15.697, expression<br>$F_{(1,24)}$ = 9.148, sipper<br>$F_{(2,48)}$ = 1.172, dose<br>$F_{(1,24)}$ = 10.663, expression X sipper	< 0.001<br>< 0.01<br>ns<br>< 0.01	**NPC**	
	B	licks (sulpiride)	control: 4 mice<br>VTA^{VGLUT2} : 10 mice	**RM 3-way ANOVA**<br>$F_{(1,24)}$ = 25.663, expression<br>$F_{(1,24)}$ = 23.641, sipper<br>$F_{(3,72)}$ = 2.233, dose<br>$F_{(1,24)}$ = 24.658, expression X sipper	< 0.001<br>< 0.001<br>ns<br>< 0.001		
	C	nosepokes<br>(counter-balanced)	VTA^{VGLUT2} :10 mice	**2-way ANOVA**<br>$F_{(1,32)}$ = 15.835, nosepoke<br>$F_{(1,32)}$ = 0.32548, drug<br>$F_{(1,32)}$ = 0.33939, nosepoke X drug	< 0.001<br>ns<br>ns		
	D	nosepokes<br>(counter-balanced)	VTA^{DAT} :5 mice	**2-way ANOVA**<br>$F_{(1,16)}$ = 39.394, nosepoke<br>$F_{(1,16)}$ = 5.3128, drug<br>$F_{(1,16)}$ = 5.0888, nosepoke X drug	< 0.001<br>< 0.05<br>< 0.05		
8		VP - TTX + 4AP	3 cells, 2 mice				
		LHb - TTX + 4AP	4 cells, 3 mice				
9		VGAT electrophysiology	VP AMPA: 2 cells, 2 mice<br>VP AMPA + DNQX: 1 cell, 1 mouse<br>VP GABA: 5 cells, 3 mice<br>VP GABA + GBZ: 4 cells, 3 mice<br>LHb AMPA:4 cells, 2 mice<br>LHb AMPA + DNQX: 1 cell, 1 mouse<br>LHb GABA:9 cells, 6 animals<br>LHb AMPA + GBZ: 3 cells, 2 mice				
10	A	Depression NAc	12 cells, 8 mice	**One Way ANOVA** - $F_{(4,59)}$ = 2.593	< 0.001	**NPC**	
	B	Depression VTA	4 cells, 3 mice				
	C	Depression LHb -Glutamate	9 cells, 6 mice	**One Way ANOVA** - $F_{(4,44)}$ = 2.606	< 0.05		
		Depression LHb - GABA	9 cells, 6 mice	**One Way ANOVA** - $F_{(4,44)}$ = 2.606	< 0.001		
	D	Depression VP	5 cells, 4 mice				
11	A	nosepokes	VTA^{DAT} : 5 mice				
	B	%nosepokes	VTA^{VGLUT2} : 9 mice				
12		%time active	control: 10 mice<br>VG2-Halo: 10 mice<br>DAT-Halo: 8 mice	**RM 2-way ANOVA**<br>$F_{(2,25)}$ = 17.72, genotype<br>$F_{(6,150)}$ = 2.4, day<br>$F_{(12,150)}$ = 4.35, day X genotype	< 0.001<br>< 0.05<br>< 0.001	**NPC**	

Supplementary Table 2. Statistical procedures used on data represented in supplemental figures.
